# No significant gender differences in driving-related skills following alcohol mixed with energy drinks during an experimental binge-drinking episode

**DOI:** 10.3389/fphar.2025.1581229

**Published:** 2025-05-23

**Authors:** Olga Hladun, Esther Papaseit, Lourdes Poyatos, Soraya Martín, Ana Pilar Pérez-Acevedo, Ana Maria Barriocanal, Tatiana Bustos-Cardona, Susana Malumbres, Rafael De La Torre, Klaus Langohr, Magí Farré, Clara Pérez-Mañá

**Affiliations:** ^1^ Department of Clinical Pharmacology, Hospital Universitari Germans Trias i Pujol and Institut de Recerca Germans Trias i Pujol (HUGTiP-IGTP), Badalona, Spain; ^2^ Department of Pharmacology, Therapeutics and Toxicology, Autonomous University of Barcelona (UAB), Cerdanyola del Vallès, Spain; ^3^ Department of Psychiatry, Hospital Universitari Germans Trias i Pujol, Badalona, Spain; ^4^ Department of Clinical Analysis and Biochemistry, Hospital Universitari Germans Trias i Pujol Badalona, Barcelona, Spain; ^5^ Integrative Pharmacology and Systems Neurosciences Research Group, Neurosciences Research Program, Hospital del Mar Medical Research Institute (IMIM), Pompeu Fabra University, Barcelona, Spain; ^6^ Department of Statistics and Operations Research, Polytechnic University of Catalonia/BarcelonaTech, Barcelona, Spain

**Keywords:** alcohol, energy drinks, binge drinking, gender difference, pharmacokinetics, male and female participants, driving-related skills

## Abstract

**Introduction:**

Consumption of alcohol mixed with energy drinks (AmEDs) is trendy among young people. It has been related to risk-taking behaviors like binge drinking and driving under the influence of alcohol. Previous data suggest that women are more sensitive to alcohol-induced impairment. The aim of the study was to assess whether women experience greater acute effects (on driving-related skills and subjective and physiological responses) after the controlled administration of alcohol and energy drinks in an experimental binge-drinking episode.

**Methods:**

A randomized, crossover, double-blind clinical trial was conducted with 28 healthy volunteers (14 men and 14 women) across four treatment conditions, namely, alcohol + energy drink (A/ED), alcohol + placebo of ED (A), placebo of alcohol + ED (ED), and both placebos (P). Men received 70 g of alcohol and women received 55 g, combined with 750 mL and 589 mL of ED, respectively; these were administered over 80 min, mimicking a binge-drinking episode. Driving-related skills (measured by a tracking test and the psychomotor vigilance task), subjective effects (using the visual analog scales (VASs, Biphasic Alcohol Effects Scale (BAES), and Addiction Research Center Inventory (ARCI)), vital signs, and alcohol and caffeine concentrations were measured over an 8-h period.

**Results:**

Peak alcohol concentrations in breath air were 0.46 mg/L in both genders, despite the alcohol dose being 21% lower in women. Similar peak blood caffeine concentrations were observed in men and women (4,500 ng/mL vs. 4,635 ng/mL with A/ED, higher than those with ED). Women reported greater drunkenness (effect size: 45 mm; 95% CI: 5–85 mm) and more alcohol-induced sedation than men (ARCI sedative subscale effect size: 12; 95% CI: 2–22), but no significant gender differences were found in driving-related skills. AmEDs slightly reduced alcohol's effects on most subjective and psychomotor outcomes, but ED did not entirely offset alcohol's effects, and no interaction between the two beverages was found for either gender.

**Conclusion:**

After a binge-drinking episode, women reported greater drunkenness and more sedation than men. Our results support that women are more sensitive to several subjective effects of alcohol, but further studies should be conducted to better elucidate gender differences in the effects of AmEDs on driving performance. ClinicalTrials.gov NCT04616859.

## 1 Introduction

Alcohol is the most widely consumed psychoactive substance globally, contributing significantly to morbidity and mortality (World Health Organization, 2024). In 2019, 22.0% of people aged 15 to 19 worldwide reported consuming alcohol in the previous year (20.5% of women and 23.5% of men), with rates reaching 41.9% and 44.0% in the Americas and Europe, respectively (World Health Organization, 2024). In Spain, the prevalence of alcohol consumption is high and more prevalent among men than women (76.4% of the general population reported alcohol consumption in the previous year, 64.5% in the last 30 days, and 9% daily in the last month) (Observatorio Español de las Drogas las Adicciones, 2023). Among adolescents (14–18 years), 58.7% of girls and 54.5% of boys reported consuming alcohol in the last 30 days in 2023 (Observatorio Español de las Drogas y las Adicciones del Ministerio de Sanidad, 2023).

There are three basic, interconnected processes through which alcohol consumption leads to health and social problems: a) the direct harmful effects of alcohol on various organs and tissues; b) the development of alcohol use disorder, which results in a drinker’s inability to regulate their consumption and frequently involves alcohol-induced mental illnesses such as depression or psychotic disorders; and c) intoxication—the immediate effects of alcohol in the hours following consumption (Babor et al., 2001). The effect of alcohol consumption is largely determined by the total volume and the pattern of consumption. Particularly harmful are the patterns associated with heavy episodic drinking (≥60 g of pure alcohol on at least one occasion at least once per month) (World Health Organization; Babor et al., 2001).

Binge drinking is a type of heavy drinking that increases blood alcohol concentrations (BACs) to 0.8 g/L (80 mg/dL; 0.08%) or higher—equivalent to 0.4 mg/L in breath alcohol concentration (BrAC); this typically corresponds to the ingestion of ≥5 drinks for men and ≥4 for women in a 2-h period (The National Institute on Alcohol Abuse and Alcoholism, 2024). Binge drinking has been associated with traffic accidents, aggression, unsafe sex, and poor academic performance (World Health Organization, 2024; López-Caneda et al., 2019). Globally, in 2019, 17% of people aged 15+ years and 38% of current drinkers engaged in heavy episodic drinking or binge drinking (World Health Organization, 2024). In Spain, the prevalence of binge drinking in 2022 was 15.4% (Observatorio Español de las Drogas las Adicciones, 2023). Furthermore, 28.2% of students reported binge drinking in the last month, with rates slightly higher in boys than in girls (Observatorio Español de las Drogas y las Adicciones del Ministerio de Sanidad, 2023).

Energy drinks (EDs) are consumed by young people with the intention of achieving increased energy, alertness, and motor and athletic performance (Clauson et al., 2008; Souza et al., 2017). Caffeine is the main ingredient, and its quantity is greater than that found in caffeinated soft drinks. Other ingredients may include guarana (a plant with high caffeine concentrations), taurine, ginseng, Ginkgo biloba, 5-hydroxytryptophan, bitter orange, B group vitamins, yerba mate, glucuronolactone, and high sugar content (Pennay et al., 2011; Wolk et al., 2012). Up to 50% of American students reported consuming EDs (CDC Healthy Schools, 2024). In Spain, consumption of EDs is very popular among adolescents (47.7% in the last 30 days), being more prevalent in boys (54.4%) than in girls (40.7%), while 14.2% of the general population reported consumption last month (Observatorio Español de las Drogas las Adicciones, 2023; Observatorio Español de las Drogas y las Adicciones del Ministerio de Sanidad, 2023).

The most commonly reported adverse effects after ED consumption are neurological (poor sleep, neuropsychiatric adverse effects, and seizures), cardiac (high blood pressure, changes in corrected QT interval (QTc), and arrhythmias), digestive and kidney disorders, metabolic adverse effects, and death (Lévy et al., 2019; Subaiea et al., 2019). Additionally, several studies point to the possibility that EDs may act as a springboard for other types of substance dependence (Higgins-Biddle and Babor, 2018; Yasuma et al., 2021).

The combined consumption of alcohol mixed with energy drinks (AmEDs) is currently a challenge to the medical community as the combination is widespread among young consumers—those more vulnerable to its effects. AmEDs are consumed to counteract alcohol’s sedative effects by allowing longer drinking sessions and to reduce the unpleasant taste of alcohol (Benson et al., 2014; Peacock et al., 2014a; McKetin et al., 2015). In the United States, in 2017, 10.6% of students (12–16 years) and 31.8% of young adults (19–28 years) drank AmEDs at least once in the past year (Johnston et al., 2021; Schulenberg et al., 2023), usually in the context of binge drinking (Emond et al., 2014). In Spain, 19.5% of students reported AmED consumption in the last 30 days, being slightly higher in boys than in girls (Observatorio Español de las Drogas y las Adicciones del Ministerio de Sanidad, 2023). In a sample of medical students at university parties (n = 527), 73.6% reported binge drinking and 12.5% reported ED consumption in the last 2 h (72.7% of the consumers of EDs mixed them with alcohol) (Farré et al., 2024).

An experimental study in animals supports that alcohol consumption with caffeine increases binge drinking (Fritz et al., 2016). In humans, AmEDs have been correlated with higher alcohol consumption (Peacock et al., 2014b; Roemer and Stockwell, 2017) and binge-drinking episodes (Graczyk et al., 2022). However, other studies showed that EDs had no impact on total alcohol consumed (Verster et al., 2016; Verster et al., 2018). Studies published to date have failed to demonstrate a reduction in drunkenness with AmEDs (Benson et al., 2014; Verster et al., 2018), while a reduction in alcohol-induced sedation and greater activation/stimulation/euphoria have been observed (Benson and Scholey, 2014; Pérez-Mañá et al., 2022). For this reason, the term wide-awake drunkenness has been used to refer to the effects of the combination of both substances (Pennay et al., 2015).

AmED consumers frequently report bad grades, use of other drugs, and driving under alcohol influence and intoxication in public (Benson and Scholey, 2014; Marczinski and Fillmore, 2014; Tucker et al., 2016). In some studies, ingesting the mix is associated with violent and risky behaviors (Peacock et al., 2014a; Roemer and Stockwell, 2017; Tucker et al., 2016). It has also been observed that EDs do not reverse alcohol-induced impairment in simple reaction time and only partially reduce alcohol’s effects in more complex tasks (McKetin et al., 2015). AmEDs predispose people to drink faster (Marczinski et al., 2017) and increase willingness to drive while intoxicated; however, EDs did not counteract alcohol’s negative impact on driving-related skills, as reported in a previous study in men (Pérez-Mañá et al., 2022). In addition, the consumption of AmEDs has been associated with an increased risk of alcohol dependence due to their stronger reinforcing properties (Marczinski and Fillmore, 2014; Lalanne et al., 2017).

Several studies have assessed the concentrations and acute effects of AmEDs in conditions that could be considered a binge-drinking episode. Peacock et al. (2015) included two administrations targeting a 0.080% BrAC, with 500 mL or 750 mL of ED, while Pérez-Mañá et al. (2022) administered 60 g of alcohol and 750 mL of ED divided into two doses; both studies were conducted in men. Alford et al. (2012) administered two alcohol doses to 10 participants of each gender (targeting 0.046% and 0.087% BrAC with one ED), but they did not compare them. Genders were compared in only one study with a limited sample size (nine men and nine women) conducted by Marczinski et al. (2012). A single alcohol dose (BrAC, 0.071%) with 3.57 mL/kg of ED was administered, and no differences among genders were found in motor coordination and subjective effects (Marczinski et al., 2012).

Alcohol concentrations and effects are higher in women than in men when the same doses are administered. Two mechanisms may account for this difference. First, differences in alcohol pharmacokinetics (Mumenthaler et al., 1999; Baraona et al., 2001): women have a lower percentage of body water than men (due to a higher % of fat), resulting in a lower distribution volume, and have lower activity of the alcohol dehydrogenase (ADH) enzyme in the stomach, so a greater percentage of the alcohol ingested would enter the bloodstream, causing a greater bioavailability of alcohol. Second, women’s central nervous systems appear to be more vulnerable to the neurotoxic effects of alcohol. These differences could, in part, be explained by sexual hormones—particularly estrogens—which modulate the dopaminergic system responsible for the reinforcing effects of alcohol (Mancinelli et al., 2016; Vinader-Caerols et al., 2017).

It has been previously shown that women display higher impairment in driving-related skills and simulated driving performance than men after alcohol administration. The study aggregated findings from 7 clinical trials, each involving 12–40 volunteers, which assessed the effects of a single alcohol dose of 0.65 g/kg. Genders had comparable BACs, close to 80 mg/dL, so it was concluded that women are more sensitive to alcohol-induced impairment. Furthermore, higher levels of subjective intoxication were also reported by women (Miller et al., 2009).

Differences in concentrations and effects of caffeine among genders are less evident. Pharmacokinetic differences have not been observed in several studies (Arnaud, 2011; Laizure et al., 2017), although it has been suggested that men can metabolize caffeine at a higher rate than women through CYP1A2 (Rasmussen et al., 2002). There are also conflicting data regarding which gender experiences stronger subjective effects from caffeine (Temple and Ziegler, 2011; Temple et al., 2015). At doses of 3–6 mg/kg of caffeine, men presented more positive and fewer negative effects than women (Domaszewski, 2023).

The study aimed to assess whether women experience higher acute effects (on driving-related skills and subjective and physiological responses) than men after the controlled administration of alcohol and energy drinks in an experimental binge-drinking episode. Furthermore, as a secondary objective, the interaction between both beverages was studied in both genders.

We did not find any other published study specifically designed to adjust the dose to achieve the same concentrations in both genders (doses are usually adjusted based on body weight, and women still have higher concentrations). To avoid the influence of these higher concentrations when comparing effects between genders, we measured concentrations of alcohol and caffeine to ensure that they were equivalent.

## 2 Materials and methods

### 2.1 Participants

Healthy male and female participants aged between 18 and 40 years, weighing between 50 and 100 kg, and with a body mass index of 20–28 kg/m^2^ were screened for enrollment in the study. To be included, participants completed a general medical evaluation, which included a 12-lead electrocardiogram (ECG), blood laboratory tests, and urine analysis.

Inclusion criteria included social alcohol consumption of at least one standard alcohol drink unit (equivalent to 10 g of alcohol in Spain) per day (average over one week), experience of binge drinking at least once per month in the past 12 months, consumption of at least 7 methylxanthine-containing drinks (such as coffee, tea, mate, cola, and EDs) per week, and previous experience with EDs. Additional inclusion criteria are provided in Supplementary Material. Women participated during the follicular phase of their menstrual cycle.

Specific exclusion criteria were as follows: personal history of drug abuse or dependence according to the Diagnostic and Statistical Manual of Mental Disorders V for any substance except nicotine. The Alcohol Use Disorders Identification Test (AUDIT) (Babor et al., 2001) was conducted, and participants with a score of 15 or higher were excluded. Female participants using hormonal contraceptives were excluded. Additional exclusion criteria are provided in Supplementary Material.

Recruitment was performed through a database of healthy volunteers from the Clinical Research Unit (UPIC) and by word of mouth at the School of Medicine.

After initial eligibility was confirmed, participants completed a training session to familiarize themselves with the psychomotor tests and questionnaires used in the study.

### 2.2 Study design

We conducted a pilot study and a definitive study. Both studies were double-blind, randomized, placebo-controlled, and crossover clinical trials in healthy volunteers.

The pilot study included seven participants (three men and four women) and three treatment conditions (alcohol + ED, alcohol + placebo ED, and placebo alcohol + ED). The goal of the pilot study was to check whether BrACs reached levels defining binge drinking for each gender and whether they were similar between genders (data and results not included in this manuscript). Each volunteer participated in three experimental sessions, separated by a washout period of at least 3 days.

In the definitive study, 14 men and 14 women were included, and all received the four treatment conditions. Participants randomly received four treatment conditions assigned using a balanced 4 × 4 Latin-square design: a) alcohol + ED (A/ED); b) alcohol + ED placebo (A); c) alcohol placebo + ED (ED); and d) alcohol placebo + ED placebo (P). Each volunteer participated in four experimental sessions separated by at least 3 days of washout period.

At the end of the definitive study, all participants had received each treatment condition, with 28 participants in each of the four treatment groups (A/ED, A, ED, and P), 14 of each gender.

Volunteers were required to abstain from drinking alcohol and beverages containing methylxanthines for 72 h before each experimental session and for up to 24 h afterward. In addition, participants followed a diet low in foods rich in taurine starting 24 h before each experimental session. Smoking was not allowed from 1 h before until the end of the experimental sessions. No additional medications were allowed from the selection visit until the end of the study (except single doses of medication that could not interfere with the objectives of the study). If an intercurrent medical process appeared, it was evaluated by the researchers to decide whether the subject should be withdrawn from the study.

### 2.3 Treatments

Alcohol and ED and their respective placebos are described in this section.

The active alcohol condition consisted of Absolut^®^ Vodka (40% ethanol by volume). Alcohol doses were selected to obtain similar alcohol concentrations in both genders based on the results of previous studies (Pérez-Mañá et al., 2022; Peacock et al., 2015; Baraona et al., 2001; Poyatos et al., 2019). In the pilot study, 50 g of alcohol was administered to women and 65 g to men, resulting in a mean peak breath alcohol concentration of 0.39 mg/L. Doses of alcohol were slightly increased in the definitive study to achieve concentrations consistent with the definition of binge drinking. The doses administered in the definitive study were 55 g for women and 70 g for men, corresponding to volumes of 172 mL and 219 mL, respectively.

The energy drink used was Red Bull^®^ The Red Edition. The doses of ED administered were 750 mL for men (3 cans of 250 mL ED, containing 240 mg of caffeine) and 589 mL for women (2.4 cans of 250 mL ED, containing 188.6 mg of caffeine). The dose of ED for men was based on our previous study (Pérez-Mañá et al., 2022). The dose for women was proportionally reduced to match the amount of alcohol administered. Caffeine doses did not exceed the maximum daily recommendations of the European Food Safety Authority (Zucconi et al., 2013) (400 mg/day or 7.5 mg/kg in a 70 kg adult).

The alcohol placebo was still water (Font Vella^®^). The ED placebo was Strawberry Fanta^®^, a beverage with a similar flavor (red fruits), color (pink), and carbonation level to the ED used in the study. The main differences between the ED and its placebo were the caffeine content (32 mg/100 mL or 80 mg per 250 mL can) and taurine content (1 g per 250 mL can).

The total volume of beverages was 761 mL for women and 969 mL for men. This volume was divided into six equal fractions; each fraction was administered every 15 min (with 5 min allocated per glass), over an 80-min period to simulate a binge-drinking episode.

### 2.4 Procedures

Participants underwent screening for coronavirus disease 2019 (COVID-19) within 24–48 h prior to each experimental session, in accordance with the hospital’s post-pandemic protocols in effect at the time. If the result was positive, they were either not included or their experimental sessions were postponed. On the day of the experimental sessions, participants arrived at UPIC at 7:45 a.m., having slept for at least 6 h and after an overnight fast of 10 h. Experimental sessions lasted 8 h from the beginning of beverage administration. Upon arrival, a urine sample was collected for drug screening using the Drug-Screen Multi 10TD Test ([Multi-Line], Ref 104101, Nal Von Minden, Germany), which tested for ten different types of drugs, namely, amphetamines, barbiturates, benzodiazepines, cocaine, 3,4-methylenedioxymethamphetamine (MDMA), methamphetamine, morphine, methadone, tricyclic antidepressants, and cannabinoids. Breath alcohol concentrations were measured using the Dräger Alcotest 5820 (Drägerwerk AG & Co, Lübeck, Germany) to confirm the absence of previous alcohol consumption. A pregnancy test (Clip Test Plus Card^®^, Ref 30701, Menarini, Spain) was performed for women. A catheter was inserted into a vein in the non-dominant arm for blood sampling.

To conceal the contents from participants and researchers, the drinks were provided cold in opaque containers with lids featuring a small opening for drinking. BrACs, blood caffeine and taurine concentrations, psychomotor effects, and subjective and physiological effects were measured over an 8-h period. At 3 h, participants were served a turkey breast sandwich and 150 mL of water as breakfast. Lunch was provided at the 5-h mark and included pasta, chicken breast with salad, an apple, and 330 mL of water. The researchers in charge of randomization, beverage preparation, and BrAC assessment did not participate in the evaluation of participants to maintain the blindness of the study. At the end of each experimental session, the volunteers were allowed to leave the unit if they had a normal physical examination and a BrAC below the legal limit for novice drivers in Spain (0.15 mg/L). Sessions for women were carried out during the same phase of the menstrual cycle (follicular phase, from day 1 to day 14) to avoid hormone interference with the results obtained (four study sessions conducted in 2 months).

### 2.5 Substance concentrations

BrACs were measured using a portable certified ethyl meter (Dräger Alcotest 5820, Drägerwerk AG & Co, Lübeck, Germany) at baseline, every 15 min during the first hour, and at 1, 2, 2.25, 2.5, 3, 4, 6, and 8 h.

Plasma concentrations of caffeine and taurine were measured at 0, 0.5, 1, 1.5, 2, 2.5, 3, 4, 6, and 8 h via liquid chromatography with tandem mass spectrometry (LC–MS–MS). Identification and quantification analyses were performed using an Agilent 1200 series HPLC system (Agilent Technologies, Wilmington, DE) coupled to a triple quadrupole mass spectrometer (6410 Triple Quad LC/MS; Agilent) with an electrospray interface. The method used has been previously described in detail by our group in a previous manuscript reporting the effects of AmEDs on driving-related skills in men (Pérez-Mañá et al., 2022). Other methods for detecting concentrations of methylxanthines or taurine are available elsewhere (Ricciutelli et al., 2014; Wang et al., 2020).

A total of 45 mL of blood was collected from each participant during each experimental session: 5 mL for baseline biochemical analysis and 4 mL for caffeine/taurine determination at 10 time points. The total blood volume collected throughout the entire study was 210 mL, including 45 mL per experimental session (4 sessions), 19 mL during the initial screening visit, and 11 mL during the final follow-up visit.

Dose-adjusted concentrations were calculated by dividing the measured concentrations by the administered dose of alcohol (70 g in men and 55 g in women), caffeine (240 vs. 180 mg), or taurine (3 g vs. 2.4 g), as appropriate.

### 2.6 Driving-related skills

To assess psychomotor performance related to driving, we included the psychomotor vigilance task (PVT), a tracking test (TT), and the Maddox Wing test. These were assessed at baseline and 1.5, 4, and 6 h after treatment administration. The training session prior to the four experimental sessions focused on psychomotor skills and consisted of 10 trials of the TT to achieve optimal performance, while the PVT was practiced on three separate occasions. Additionally, the Maddox wing was performed twice.

The PVT is a validated test that can be reliably extrapolated to assess driving-related skills (Jongen et al., 2014). It uses software (PC-PVT 2.0 for Windows 10 OS) and a laptop to measure reaction time by presenting a known (numeric) stimulus at a known location (center of the computer screen) to elicit a known response (pressing the mouse button). The only uncertainty lies in the timing of the stimulus, which appears at a variable interval between the test response and the next stimulus (Khitrov et al., 2014; Reifman et al., 2018) The task duration was defined to be 5 min (Sylvia et al., 2004). The outcomes measured were as follows: mean latency (the mean response times for all trials, in ms), median latency (median reaction time, in ms), and SD latency (standard deviation of reaction time, in ms) (Sylvia et al., 2004; Rohsenow et al., 2010).

The TT is an adaptation of the classic Critical Tracking Task (Jex et al., 1966) and is similar to the Visuomotor Bimanual Coordination Test used to obtain a driving license in Spain (Gombao et al., 2006). In this interactive task, participants are asked to keep two yellow vehicles, displayed on the screen, centered on their respective lanes using controls (joysticks). The duration of the test is 290 s, and it is also performed on a laptop. The outcomes measured were as follows: the total time a vehicle was outside the road (time out), the number of errors (occasions where a car was outside the road for more than 0.4 s), and the number of gyres (changes in the joystick direction). The tracking test used in this study was the same as that in our previous trial with AmEDs in men (Pérez-Mañá et al., 2022), where we demonstrated that impairment in the test correlated with the administered dose of alcohol. Other tracking tests have also been used to assess driving-related skills after the consumption of psychopharmacological drugs as they can provide supportive information that complements findings from on-road assessments (Verster and Roth, 2012). The main outcome of the study was changes in the mean latency of the PVT.

The Maddox Wing device measures heterophoria due to extraocular muscle imbalance. It quantifies exophoria as an indicator of relaxation of the extraocular muscles and esophoria as an indicator of the contraction of the extraocular muscles. It employs dissociated vision to project a movable arrow over a calibrated ruler, allowing the measurement of ocular deviations. A leftward shift (even numbers) indicates exophoria, typically linked to muscle relaxation and observed after sedative use, while a rightward shift (odd numbers) indicates esophoria, associated with muscle contraction and observed after stimulant intake such as amphetamines or MDMA (Camí et al., 2000; Peiró et al., 2013; Farré et al., 2015; Papaseit et al., 2016). One participant was excluded from the placebo group for these outcomes. Comparisons among treatments were conducted with 13 male participants in the placebo group. For the time-course analysis, data from 13 male participants were included across all treatment conditions.

### 2.7 Subjective effects’ rating scales

Subjective effects were evaluated using visual analog scales (VASs) (Peiró et al., 2013; Farré et al., 2015; Ménétrey et al., 2005), the Biphasic Alcohol Effects Scale (BAES) (Martin et al., 1993), and the Spanish-validated version of the short-form Addiction Research Center Inventory (ARCI) (Farré et al., 2015; Lamas et al., 1994).

VASs consist of a 100 mm (0–100 mm) straight line with the word “none/nothing” at one end and the word “extremely” at the other. The effects rated were drunkenness, dizziness, drowsiness, heart palpitations, anxiety, and headache, assessed at baseline and 0.5, 1, 1.5, 2, 2.5, 3, 4, 6, and 8 h post-treatment. Capability to drive (EAVc1) and willingness to drive under different circumstances (EAVc2: willingness to drive an ill child to the hospital; EAVc3: willingness to drive a sick friend home; and EAVc4: willingness to drive a friend to a party) were measured using a VAS at baseline and 1, 1.5, 4, 6, and 8 h post-treatment. Additionally, a VAS measuring “like the drug” and “desire to continue drinking” was administered at 1.5 h.

The BAES identifies the stimulant and sedative effects of alcohol over time using a 14-adjective rating scale (7 adjectives for stimulation and 7 for sedation). A Likert scale ranging from 0 (not at all) to 10 (extremely) is used for each adjective and was measured at baseline and 1, 1.5, 2, 3, 4, 6, and 8 h post-treatment. BAES activation/stimulation (BAES-A) is the sum of the scores of the seven activation items, namely, euphoric, fully energetic, excited, mentally stimulated, talkative, lively, and vigorous. Each activation item is scored from 0 to 10 (BAES-A scores from 0 to 70). BAES sedation (BAES-S) is the sum of the scores of the seven sedation items, namely, difficulty in concentrating, discouraged, heavy-headed, sluggish, sedated, slow thinking, and I feel slow. Each sedation item is scored from 0 to 10 (BAES-S scores range from 0 to 70).

A validated Spanish version of the 49-item short-form Addiction Research Center Inventory (ARCI) was used at baseline and 1, 1.75, 4, 6, and 8 h post-treatment. The ARCI contains 49 true/false items grouped into five subscales: PCAG (pentobarbital–chlorpromazine–alcohol group, measures sedation), MBG (morphine–benzedrine group, measures euphoria), LSD (lysergic acid diethylamide group, measures dysphoria), BG (measures intellectual efficiency), and A (a scale sensitive to the effects of d-amphetamine). The PCAG scale is sensitive to the effects of alcohol, while the MBG, BG, and A scales are sensitive to psychostimulants like caffeine.

The Drink Identification Questionnaire was administered at 8 h post-treatment. Participants were asked to select the beverage combination that best described the one they received during each session.

Dose-adjusted effects were calculated for the most important outcomes (mean latency, time out, drunkenness, and BAES) by dividing the results obtained by the alcohol dose administered.

### 2.8 Vital signs

Systolic and diastolic blood pressure, heart rate, oral temperature, and pupil diameter were all measured at baseline (−30 and −15 min) and 0.5, 1, 1.5, 2, 2.5, 3, 4, 6, and 8 h after the start of beverage administration. They were measured using a vital signs monitor (Philips Sure Signs VM4 monitors, Philips, Amsterdam, Netherlands). The electrocardiogram was continuously monitored during the sessions for safety reasons with the same monitor. The Haab pupil gauge was used to measure pupil diameter under constant lighting.

### 2.9 Tolerability of the drinks and adverse events

The tolerability of the drinks was assessed during the experimental sessions, and any spontaneously reported adverse events were collected throughout the study.

### 2.10 Ethical aspects

The Human Research Ethics Committee at Hospital Germans Trias i Pujol approved the study protocol and participant information sheet and consent (CEIm-HUGTiP, approval number: PI-19–144). The study was carried out in accordance with the Declaration of Helsinki and Spanish clinical research regulations and was registered on ClinicalTrials.gov (NCT04616859).

### 2.11 Statistical analysis

Data from one man in the placebo condition for driving-related skills and another in the taurine concentrations were discarded due to outlier results based on the Dixon test.

Differences from baseline and the following pharmacokinetic parameters were calculated for all outcomes: area under the curve of the concentrations and effects from 0 to 8 h (AUC), the time needed to reach the maximum concentration and effect (t_max_), and the maximum/peak concentration (C_max_) and effect (peak or E_max_).

C_max_ and AUC of alcohol, caffeine, and taurine concentrations were analyzed using two-way ANOVA models with repeated measures that included treatment (A/alcohol + ED or alcohol + ED/ED), gender, and treatment–gender interactions as independent factors. Based on these models, treatments were compared separately for both women and men. Given that none of the interactions were statistically significant, the interaction term was removed from all models, and both overall mean treatment differences and mean gender differences were computed along with the corresponding 95% confidence intervals. Additionally, for each concentration, the t_max_ values between both treatments were compared separately for women and men using the nonparametric Wilcoxon test.

For E_max_ and AUC of subjective, psychomotor, and physiological parameters, all possible comparisons between study groups were performed using three-way ANOVA models with repeated measures. These models included alcohol (active and placebo), ED (active and placebo), and gender, as well as all two-way and three-way interactions. In these models, the alcohol*ED interaction can be interpreted as indicative of the capacity of ED to affect the alcohol effect among both women and men. Comparisons of interest included estimating both the alcohol and ED effects (vs. placebo) and comparing alcohol versus alcohol + ED, all performed separately for women and men. Given this multiplicity of post-hoc comparisons, confidence intervals and *p*-values were computed based on the multivariate t-distribution of the vector of test statistics (Hothorn et al., 2008) to ensure a family-wise error rate of 0.05. The comparisons of interest are those reported in the Results section, along with comparisons among genders in A and A/ED conditions.

Furthermore, the time course (T-C) of concentrations and effects between treatment conditions was compared using a three-way repeated measures ANOVA model that included treatment, time, and gender as factors, as well as all two- and three-way interactions. Adjustment for multiple gender comparisons across treatment conditions at each time was made using the Bonferroni correction. In the Results section, the differences reported are as follows: A vs. A/ED or differences with placebo for all conditions (see figures). The table in Supplementary Material reports all comparisons between treatments.

Repeated-measures correlations were calculated to assess within-individual correlations between alcohol concentrations and effects across the different time points separately for each gender (Bakdash and Marusich, 2017).

The analyses were performed using PAWS Statistics version 18 (SPSS Inc., Chicago, IL, USA) and R Statistical Software version 4.3.2 (Vienna, Austria; https://www.r-project.org/). A *p*-value of less than 0.05 was considered statistically significant.

## 3 Results

### 3.1 Study participants

A total of 28 healthy volunteers participated in the definitive study, 14 men and 14 women. They had a mean age of 22.18 years old (SD: 1.44) and a mean weight of 68.38 kg (SD: 8.72), and their BMI was 23.04 (SD: 2.74). They reported recreational alcohol consumption of 20.45 g/day (SD: 10.61) and 2.78 caffeine-containing beverages per day. All of them reported binge-drinking episodes (1.93 episodes/month) and previous ED consumption (3.14 ED/last month).

In our sample, men weighed more (73.64 vs. 63.12 kg) and were taller (179.51 vs. 165.44 cm) than women, whereas the differences in BMI and both daily alcohol and caffeine consumption were minimal (data not shown).

### 3.2 Concentrations

The alcohol-administered dose was 0.96 mg/kg (SD: 0.07) in men and 0.88 mg/kg (SD: 0.10) in women; the caffeine dose was 3.3 mg/kg (SD: 0.25) in men and 3.00 mg/kg (SD: 0.35) in women; and finally, taurine doses were 41.00 mg/kg (SD: 3.11) and 38.50 mg/kg (SD: 4.44), respectively.

**FIGURE 1:**
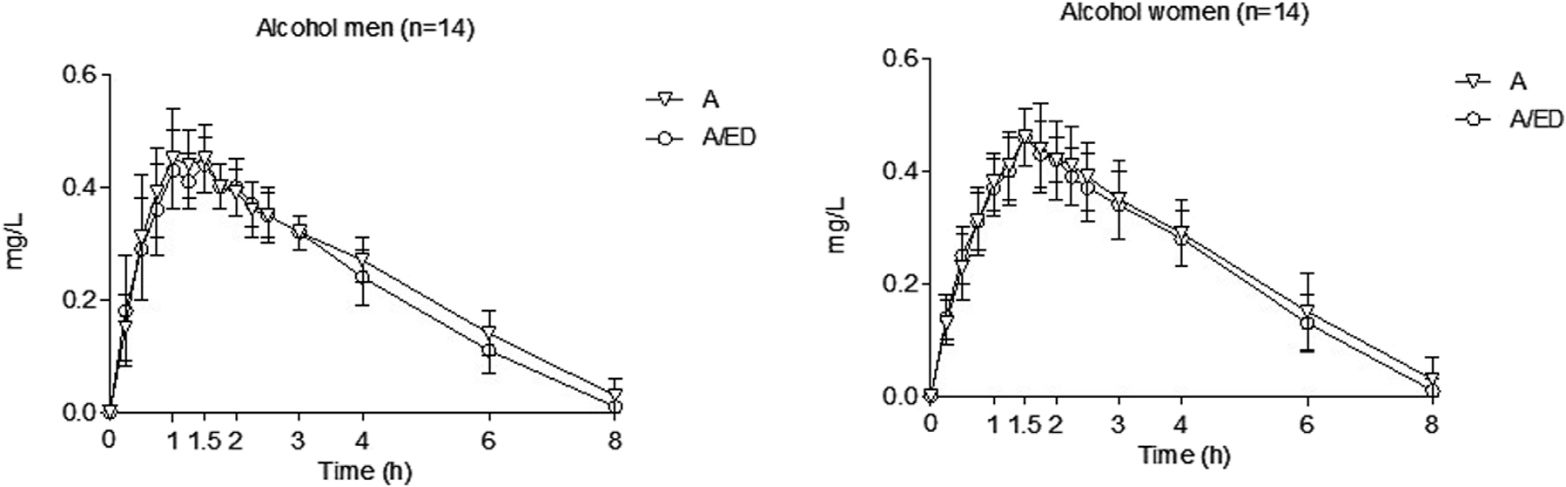
Alcohol concentrations in breath air. Data points and error bars represent the mean and SD values. A, alcohol + energy drink placebo; A/ED, alcohol + energy drink. Differences were analyzed using a repeated measures ANOVA, with treatment and time as factors.

**FIGURE 2:**
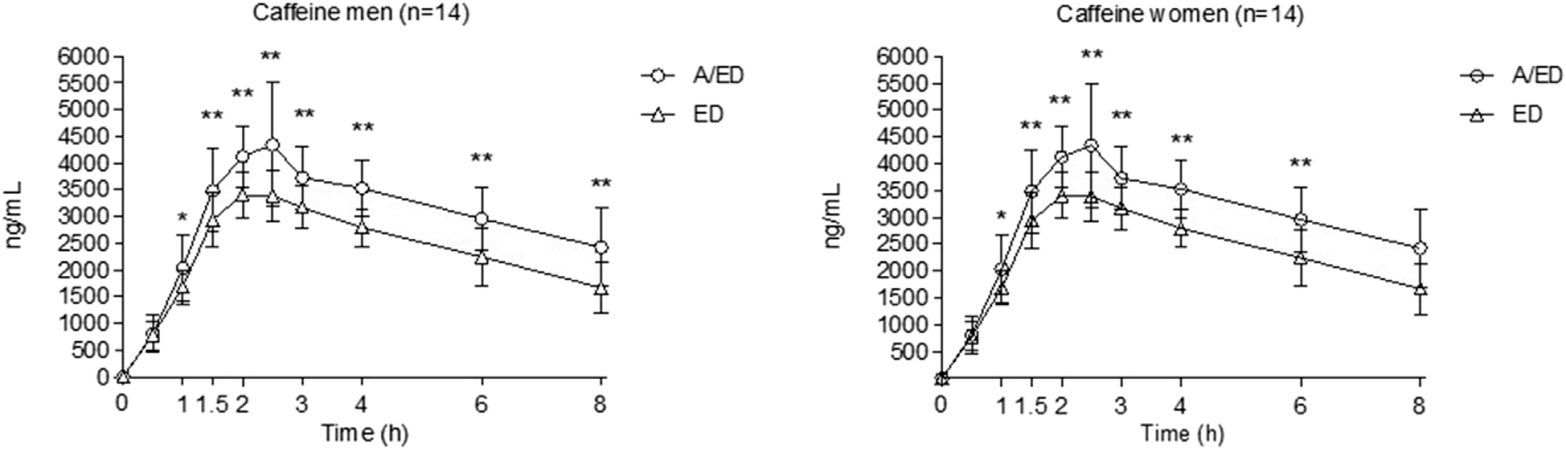
Caffeine plasma concentrations. Data points and error bars represent the mean and SD values. A/ED, alcohol + energy drink; ED, energy drink + placebo alcohol. *p < 0.05; **p < 0.01. Differences were analyzed using a repeated measures ANOVA, with treatment and time as factors.

Pharmacokinetic parameters and effect sizes among treatment conditions are shown in Table 1, separated by gender. Furthermore, Figures 1, 2 present the time courses of alcohol and caffeine concentrations. Alcohol concentrations in breath air reached more than 0.4 mg/L, corresponding to a blood alcohol concentration (BAC) of 80 mg/dL (0.8 g/L or 0.08%), which meets the criteria for the definition of an experimental binge-drinking episode.

**TABLE 1:** Concentrations of alcohol, caffeine, and taurine.

Variable	Gender	A or ED Mean ± SD	A/ED Mean ± SD	Parameter	*p*
Alcohol in breath air	Men	0.47 ± 0.07	0.46 ± 0.06	C_max_ (mg/L)	0.994
		1.13	1.13	T_max_ (h)	0.810
		1.90 ± 0.29	1.79 ± 0.24	AUC_0–8h_ (mg.h/L)	0.127
	Women	0.46 ± 0.06	0.46 ± 0.05	C_max_ (mg/L)	0.998
		1.50	1.50	T_max_ (h)	0.577
		1.98 ± 0.43	1.90 ± 0.33	AUC_0–8h_ (mg.h/L)	0.349
Dose-adjusted alcohol in breath air	Men	0.007 ± 0.001	0.007 ± 0.001	C_max_ (mg/L/g)	0.994
		0.027 ± 0.004	0.026 ± 0.003	AUC_0–8h_ (mg.h/L/g)	0.187
	Women	0.008 ± 0.001	0.008 ± 0.001	C_max_ (mg/L/g)	0.997
		0.036 ± 0.008	0.035 ± 0.006	AUC_0–8h_ (mg.h/L/g)	0.256
Caffeine plasma	Men	3,501 ± 411	4,500 ± 1,064	C_max_ (ng/mL)	0.000
		2.25	2.00	T_max_ (h)	0.763
		18,847 ± 3,208	23,861 ± 3,652	AUC_0–8h_ (ng.h/mL)	0.000
	Women	3,913 ± 628	4,635 ± 562	C_max_ (ng/mL)	0.001
		2.00	2.50	T_max_ (h)	0.234
		21,455 ± 4,508	26,121 ± 4,074	AUC_0–8h_ (ng.h/mL)	0.000
Dose-adjusted caffeine plasma	Men	14.6 ± 1.71	18.8 ± 4.43	C_max_ (ng/mL/mg)	0.000
		78.5 ± 13.4	99.4 ± 15.2	AUC_0–8h_ (ng.h/mL/g)	0.000
	Women	20.7 ± 3.32	24.5 ± 2.97	C_max_ (ng/mL/mg)	0.000
		114 ± 23.9	138 ± 21.6	AUC_0–8h_ (ng.h/mL/g)	0.000
Taurine plasma	Men	75,200 ± 18,310	81,856 ± 17,737	C_max_ (ng/mL)	0.158
		2.00	2.00	T_max_ (h)	0.564
		236,315 ± 61,287	241,292 ± 65,542	AUC_0–8h_ (ng.h/mL)	0.890
	Women	87,407 ± 18,436	86,903 ± 12,810	C_max_ (ng/mL)	0.989
		2.00	2.00	T_max_ (h)	0.157
		265,414 ± 65,613	260,761 ± 39,705	AUC_0–8h_ (ng.h/mL)	0.875
Dose-adjusted taurine plasma	Men	25,067 ± 6,103	27,285 ± 5,912	C_max_ (ng/mL/g)	0.270
		78,772 ± 20,429	80,431 ± 21,847	AUC_0–8h_ (ng.h/mL/g)	0.922
	Women	36,420 ± 7,682	36,210 ± 5,337	C_max_ (ng/mL/g)	0.988
		110,589 ± 27,339	108,650 ± 16,544	AUC_0–8h_ (ng.h/mL/g)	0.867

n = 14 men in all conditions (except n = 13 in taurine for A/ED and ED conditions) and n = 14 women in all conditions. A, alcohol + placebo energy drink; A/ED, alcohol + energy drink; AUC_0–8 h_, area under the curve of concentrations from 0 until 8 h; C_max_, maximum or peak concentrations; ED, energy drink + placebo alcohol; SD, standard deviation; t_max_, time to reach peak concentrations.

Alcohol concentrations peaked at 1.13–1.50 h and were undetectable for most volunteers by 8 h. Based on raw data, when breath alcohol concentrations were compared between women and men, no differences were found (peak, AUC). When alcohol concentrations were adjusted for the administered dose, women had higher C_max_ (gender effect: men vs. women, −0.002 mg/L/g; 95% CI: −0.003 to −0.001; *p* < 0.001) and AUCs (gender effect: −0.009 mg x h/L x g; 95% CI: −0.013 to −0.004; *p* < 0.001) than men. No differences were observed between the conditions (A/ED vs. A) for C_max_, AUC, and t_max_ in each gender, including when concentrations were dose-adjusted. Globally, energy drinks slightly reduced the AUC of alcohol concentrations (ED effect: −0.095 mg x h/L; 95% CI: −0.185 to −0.005; *p* < 0.05), even when dose-adjusted (ED effect: −0.002 mg × h/L/g; 95% CI: −0.003 to −0.000; *p* < 0.05).

Differences between genders were found at individual time points. In the A condition, men had higher concentrations than women at 0.5, 0.75, and 1 h, while women had higher concentrations at 2.5 h. In the condition A/ED, men had higher concentrations at 1 h, while the opposite was observed at 4 h.

Caffeine concentration peaked at 2–2.5 h and remained at half the peak value at 8 h. No statistically significant differences were found between men and women. However, when caffeine concentrations were adjusted to the caffeine-administered dose, C_max_ (gender effect: −5.95 ng/mL; 95% CI: −8.34–83.55; p < 0.001) and AUC of concentrations (−36.89 ng x mg/mL; 95% CI: −52.31 to −21.47; p < 0.001) were higher in women than in men. Caffeine concentrations were higher when alcohol was present in the beverage in both genders (alcohol effect: 861.107 ng/mL; 95% CI: 547.95 to 1,174.27; p < 0.001 for C_max_ and 4,840.16 mg/L x h; 95% CI: 3,874.09 to 5,806.23; p < 0.001 for AUC), and this effect remained significant even after when dose adjustment (alcohol effect: 3.99 ng/mL; 95% CI: 2.66 to 5.33; p < 0.001 for C_max_ and 22.80 ng x h/mL; 95% CI 18.21 to 27.70; p < 0.001 for AUC).

Regarding taurine, one man was excluded from analysis due to outlier data (peak and AUC), and two in the T-C of concentrations based on the Dixon test. Taurine concentrations peaked at 2 h and nearly returned to baseline 8 h after administration. There were no differences between A/ED vs. ED conditions and no gender differences in C_max_ or AUC. When concentrations were adjusted to the taurine-administered dose, C_max_ (gender effect: −10,062.64 ng/mL; 95% CI: −14,896.29 to 5,229.00; p < 0.001) and AUC (gender effect: −29,449.96 ng × g/mL; 95% CI: −46,798.91 to 12,101.00; p < 0.001) were again higher in women than in men.

### 3.3 Effects

Alcohol in both conditions (A and A/ED) produced its prototypical effects. Alcohol induced drunkenness, drowsiness, and dizziness; increased reaction time in PVT and time spent outside the road; produced biphasic effects (stimulation–sedation), depending on the time elapsed from the administration; reduced subjective capability and willingness to drive; increased euphoria, sense of wellbeing, and sedation in ARCI; and increased the heart rate.

A reduction in the effects of alcohol with EDs was observed in several subjective and performance outcomes. Differences between the A and A/ED conditions reached statistical significance in mean and median latency (PVT), number of errors (TT), headache, and vital signs. ED had statistically significant effects only on mean latency and systolic/diastolic blood pressure. Interacting effects between ED and alcohol that were statistically significant were not observed, except for headache, diastolic blood pressure, and heart rate.

Alcohol and ED peak and AUC values for all outcomes and conditions in each gender are reported in Tables 2–4. Tables also include effect sizes for alcohol and ED (differences from placebo), the comparison of more interest between conditions (A/ED vs. A), and the *p*-value of the interaction between alcohol and ED. Details regarding time-point comparisons between conditions and genders are provided in Table 1 in Supplementary Material.

**TABLE 2:** Results of driving-related skills.

Outcome	Gender	A Mean ± SD	A/ED Mean ± SD	ED Mean ± SD	*p* Mean ± SD	Parameter	ES (95% CI) A	ES (95% CI) ED	ES (95% CI) A/ED vs. A	Interaction
Number of gyres (TT)	Men	119.80 ± 149.50	113.07 ± 79.52	43.14 ± 109.91	12.77 ± 147.67	Peak	106 (−44.67, 257.81)	30.08 (−121.01, 181.17)	−6.71 (−154.92, 141.49)	N.S.
		379.50 ± 502.00	342.40 ± 279.9	159.07 ± 411.05	84.90 ± 312.30	AUC_0–6 h_	294.174 (−113.79, 702.14)	76.75 (−334.22, 481.71)	−37.10 (−437.14, 362.95)	N.S.
	Women	77.60 ± 161.00	136.79 ± 208.82	30.29 ± 129.02	17.00 ± 103.41	Peak	60.64 (−87.56, 208.85)	13.29 (−134.92, 161.49)	59.14 (−89.06, 207.35)	N.S.
		221.90 ± 405.00	427.33 ± 431.43	126.02 ± 374.98	85.71 ± 304.30	AUC_0–6 h_	136.14 (−263.90, 536.18)	40.30 (−359.74, 440.35)	205.47 (−194.57, 605.52)	N.S.
Time spent outside the road (TT)	Men	9.12 ± 8.45	2.88 ± 6.00	−1.66 ± 3.26	1.84 ± 4.31	Peak	7.28 (0.75, 13.81)*	−3.51 (−10.04, 3.02)	−6.24 (−12.65, 0.17)	N.S.
		24.15 ± 19.61	8.20 ± 14.48	−5.44 ± 10.59	3.57 ± 12.81	AUC_0–6 h_	20.58 (3.89–37.27)**	−9.01 (−25.71, 7.68)	−15.95 (−32.33, 0.43)	N.S.
	Women	5.01 ± 6.66	3.90 ± 6.46	−3.14 ± 5.21	0.44 ± 6.30	Peak	4.57 (−1.84, 10.98)	−3.58 (−9.99, 2.84)	−1.11 (−7.52, 5.92)	N.S.
		13.41 ± 16.30	9.73 ± 16.32	−8.94 ± 15.18	−2.77 ± 16.16	AUC_0–6 h_	16.18 (3.89, 37.27)**	−6.16 (−22.55, 10.22)	−3.68 (−20.06, 12.70)	N.S.
Dose-adjusted time spent outside the road s/g (TT)	Men	0.13 ± 0.12	0.04 ± 0.08	−0.02 ± 0.05	0.02 ± 0.06	Peak	0.11 (−0.00, 0.21)	−0.05 (−0.16, 0.06)	−0.09 (−0.20, 0.02)	N.S.
		0.35 ± 0.28	0.12 ± 0.21	−0.08 ± 0.15	0.05 ± 0.18	AUC_0–6 h_	0.29 (0.02, 0.57)*	−0.13 (−0.41, 0.15)	−0.23 (−0.50, 0.04)	N.S.
	Women	0.09 ± 0.12	0.07 ± 0.12	−0.06 ± 0.10	0.01 ± 0.11	Peak	0.08 (−0.02, 0.19)	−0.07 (−0.17, 0.04)	−0.02 (−0.13, 0.09)	N.S.
		0.24 ± 0.30	0.18 ± 0.30	−0.16 ± 0.28	−0.05 ± 0.29	AUC_0–6 h_	0.30 (0.03, 0.57)*	−0.11 (−0.38, 0.16)	−0.07 (−0.34, 0.21)	N.S.
Errors (TT)	Men	10.64 ± 10.87	2.36 ± 6.99	−1.79 ± 3.26	2.77 ± 5.05	Peak	7.72 (0.57, 14.87)+	−4.71 (−11.86, 2.44)	−8.29 (−15.29, −1.29)*	N.S.
		28.34 ± 25.12	8.63 ± 16.56	−5.32 ± 8.88	5.15 ± 12.68	AUC_0–6 h_	23.05 (4.79, 41.32)**	−10.61 (−28.88, 7.65)	−19.71 (−37.60, −1.81)*	N.S.
	Women	4.14 ± 9.17	3.71 ± 8.35	−2.50 ± 5.36	−1.07 ± 4.75	Peak	5.21 (−1.79, 14.87)	−1.43 (−8.43, 5.57)	−0.43 (−7.43, 6.57)	N.S.
		18.23 ± 19.32	12.26 ± 17.07	−10.63 ± 20.94	−2.84 ± 15.77	AUC_0–6 h_	21.07 (3.18, 38.97)*	−7.79 (−25.68, 10.11)	−5.97 (−23.87, 11.92)	N.S.
Mean latency ms (PVT)	Men	71.34 ± 29.93	35.78 ± 32.80	−13.32 ± 30.46	−0.29 ± 24.15	Peak	7.39 (43.52, 99.25)***	−13.28 (−41.14, 14.59)	−35.57 (−62.84, −8.30)**	N.S.
		236.44 ± 108.85	121.48 ± 104.47	−48.48 ± 120.50	−3.06 ± 79.27	AUC_0–6 h_	239.86 (138.00, 341.72)***	−45.05 (−146.91, 56.80)	−114.96 (−214.70, −15.21)*	N.S.
	Women	71.54 ± 28.88	50.28 ± 31.42	−17.48 ± 24.78	12.32 ± 24.40	Peak	59.23 (31.95, 86.50)***	−29.80 (−57.07, −2.52)*	−21.26 (−48.54, 6.01)	N.S.
		267.36 ± 117.76	142.69 ± 96.21	−61.06 ± 93.29	37.31 ± 83.08	AUC_0–6 h_	230.05 (130.30–329.79)***	−98.37 (−198.11, 1.38)	−124.67 (−224.42, −24.93)**	N.S.
Dose-adjusted mean latency ms/g (PVT)	Men	1.02 ± 0.43	0.51 ± 0.0.47	−0.19 ± 0.44	−0.00 ± 0.34	Peak	1.02 (0.57, 1.47)***	−0.19 (−0.64, 0.26)	−0.51 (−0.95, −0.01)*	N.S.
		3.38 ± 1.55	1.76 ± 1.49	−0.69 ± 1.72	0.45 ± 1.13	AUC_0–6 h_	3.43 (1.76, 5.09)***	−0.64 (−2.31, 1.02)	−1.64 (−3.27, −0.01)*	N.S.
	Women	1.30 ± 0.52	0.92 ± 0.57	−0.32 ± 0.45	0.23 ± 0.45	Peak	1.08 (0.63, 1.52)***	−0.54 (−0.99, −0.10)**	−0.39 (−0.83, 0.06)	N.S.
		4.87 ± 2.14	2.60 ± 1.75	−1.11 ± 1.70	0.68 ± 1.51	AUC_0–6 h_	4.18 (2.55, 5.81)***	−1.79 (−3.42, −0.16)**	−2.27 (−3.90, −0.63)**	N.S.
SD latency ms (PVT)	Men	48.03 ± 46.34	40.11 ± 85.32	3.20 ± 62.24	2.29 ± 34.04	Peak	45.55 (−7.51, 98.61)	0.72 (−52.34, 53.79)	−7.92 (−59.88, 44.04)	N.S.
		111.97 ± 103.91	99.68 ± 220.17	−2.02 ± 219.49	−2.33 ± 118.13	AUC_0–6 h_	114.14 (−43.17, 271.45)	0.14 (−157.17, 157.45)	−12.29 (−166.35, 141.77)	N.S.
	Women	32.58 ± 32.20	27.59 ± 57.37	−3.46 ± 43.20	17.92 ± 43.47	Peak	14.66 (−37.29, 66.62)	−21.38 (−73.34, 30.58)	−4.99 (−56.95, 46.97)	N.S.
		85.22 ± 97.91	56.19 ± 191.77	−29.85 ± 136.36	24.70 ± 89.23	AUC_0–6 h_	60.52 (−93.54, 214.58)	−54.55 (−208.62, 99.51)	−29.03 (−183.09, 125.03)	N.S.
Median latency ms (PVT)	Men	60.93 ± 25.23	39.11 ± 25.06	−15.11 ± 22.94	1.08 ± 22.66	Peak	58.89 (30.82, 88.95)***	−16.15 (−45.21, 12.91)	−21.82 (−50.32, 6.68)	N.S.
		208.94 ± 97.93	117.13 ± 97.38	−57.28 ± 72.90	7.88 ± 69.57	AUC_0–6 h_	201.70 (110.95, 292.45)***	−64.52 (−155.27, 26.23)	−91.80 (−180.69, −2.92)*	N.S.
	Women	67.07 ± 28.36	31.93 ± 41.44	−11.68 ± 25.56	13.04 ± 21.87	Peak	54.04 (25.53, 82.54)***	−24.71 (−53.22, 3.79)	−35.14 (−63.65, −6.64)**	N.S.
		255.02 ± 118.22	108.32 ± 88.72	−41.20 ± 85.93	36.16 ± 75.65	AUC_0–6 h_	218.86 (129.98, 307.74)***	−77.36 (−166.24, 11.52)	−146.70 (−235.58, −57.82)***	N.S.
Maddox wing	Men	1.11 ± 1.36	1.29 ± 0.70	0.29 ± 0.58	0.25 ± 0.510	Peak	0.86 (0.08–1.64)*	0.04 (−0.75–0.82)	0.18 (−0.60, 0.96)	N.S.
		4.47 ± 5.37	4.73 ± 3.29	0.90 ± 2.00	0.83 ± 1.42	AUC_0–6 h_	3.64 (0.73, 6.54)**	0.07 (−2.84, 2.98)	0.27 (−2.64, 3.18)	N.S.
	Women	1.32 ± 0.78	1.21 ± 0.80	0.18 ± 0.58	−0.07 ± 0.55	Peak	1.39 (0.61, 2.17)***	0.25 (−0.53, 1.03)	−0.11 (−0.89, 0.67)	N.S.
		4.37 ± 2.69	4.18 ± 3.38	0.42 ± 1.69	0.58 ± 1.29	AUC_0–6 h_	3.79 (0.88, 6.69)**	−0.16 (−3.07, 2.75)	−0.19 (−3.10, 2.72)	N.S.

Data of one male volunteer under the placebo condition were not included due to outlier results. A, alcohol + placebo energy drink; A/ED, alcohol + energy drink; ED, energy drink + placebo alcohol; AUC_0–6 h_, area under the curve of effects from 0 until 6 h; CI, confidence interval; ES, effect size; P, placebo alcohol + placebo energy drink; PVT, psychomotor vigilance task; TT, tracking test; SD, standard deviation; N.S., not significant; p < 0.05; **p < 0.01; ***p < 0.001. n = 14 men in all conditions (except n = 13 in placebo for PVT and TT outcomes) and n = 14 women in all conditions.

**TABLE 3:** Results of subjective effects.

Outcome	Gender	A Mean ± SD	A/ED Mean ± SD	ED Mean ± SD	*p* Mean ± SD	Parameter	ES (95% CI) A	ES (95% CI) ED	ES (95% CI) A/ED vs. A	Interaction
Drunkenness mm	Men	42.43 ± 19.93	43.36 ± 21.02	0.36 ± 1.34	0.14 ± 0.36	Peak	42.29 (28.64, 55.93)***	0.21 (−13.43–13.86)	0.93 (−12.72–14.57)	N.S.
		75.34 ± 39.94	83.00 ± 51.88	0.18 ± 0.67	0.25 ± 0.64	AUC_0–8 h_	75.09 (35.50, 114.68)***	−0.07 (−39.66, 39.51)	7.66 (−31.93, 47.25)	N.S.
	Women	47.79 ± 18.82	44.07 ± 22.38	0.36 ± 1.34	1.21 ± 4.54	Peak	46.57 (32.93, 60.22)***	−0.85 (−14.50, 12.79)	−3.71 (−17.36, 9.93)	N.S.
		120.16 ± 76.82	101.09 ± 63.44	0.30 ± 1.14	1.25 ± 4.68	AUC_0–8 h_	118.91 (79.35, 158.50)***	−0.95 (−40.53, 38.64)	−19.07 (−58.66, 20.51)	N.S.
Dose-adjusted drunkenness mm/g	Men	0.61 ± 0.28	0.62 ± 0.30	0.00 ± 0.02	0.00 ± 0.00	Peak	0.61 (0.38, 0.83)***	0.00 (−0.22, 0.23)	0.01 (−0.21, 0.24)	N.S.
		1.07 ± 0.57	1.19 ± 0.76	0.00 ± 0.01	0.00 ± 0.01	AUC_0–8 h_	1.07 (0.40, 1.75)***	−0.00 (−0.68, 0.67)	0.11 (−0.57, 0.79)	N.S.
	Women	0.87 ± 0.34	0.80 ± 0.41	0.01 ± 0.02	0.02 ± 0.08	Peak	0.85 (0.62, 1.07)***	−0.02 (−0.24, 0.21)	−0.07 (−0.29, 1.55)	N.S.
		2.18 ± 1.39	1.83 ± 1.15	0.00 ± 0.02	0.23 ± 0.85	AUC_0–8 h_	2.16 (1.49, 2.84)***	−0.02 (−0.69, 0.66)	−0.35 (−1.02, 0.33)	N.S.
Drowsiness mm	Men	28.29 ± 25.83	25.29 ± 26.36	4.36 ± 7.02	7.86 ± 11.79	Peak	20.43 (1.24, 39.62)*	−3.5 (−22.69, 15.69)	−3.00 (−22.19, 16.19)	N.S.
		72.48 ± 83.48	64.05 ± 67.11	7.41 ± 14.84	10.48 ± 19.80	AUC_0–8 h_	62.00 (0.76, 124.76)	−3.07 (−65.83, 59.69)	−8.43 (−71.19, 54.33)	N.S.
	Women	36.71 ± 26.73	29.36 ± 29.34	3.21 ± 5.56	15.14 ± 20.34	Peak	21.57 (2.38, 40.77)*	−11.93 (−31.12, 7.27)	−7.36 (−26.55, 11.84)	N.S.
		131.32 ± 119.42	83.13 ± 96.63	4.41 ± 8.36	37.21 ± 58.66	AUC_0–8 h_	94.11 (31.35, 156.87)***	−32.80 (−95.56, 29.95)	−48.20 (−110.95, 14.56)	N.S.
Dizziness mm	Men	22.43 ± 28.40	16.79 ± 21.79	0.64 ± 2.13	0.36 ± 1.34	Peak	22.07 (5.05, 39.09)**	0.29 (−16.73, 17.30)	−5.64 (−22.66, 11.38)	N.S.
		42.86 ± 63.93	25.82 ± 39.59	0.68 ± 2.40	0.32 ± 1.20	AUC_0–8 h_	42.54 (−2.6, 87.67)	0.36 (−44.79, 45.92)	−17.04 (−62.17, 28.10)	N.S.
	Women	26.14 ± 21.31	31.07 ± 29.73	0.86 ± 1.88	0.93 ± 3.47	Peak	25.21 (8.20, 42.23)***	−0.07 (−17.08, 16.95)	4.93 (−12.09, 21.95)	N.S.
		68.39 ± 76.93	68.77 ± 87.30	0.84 ± 1.82	1.14 ± 4.28	AUC_0–8 h_	67.25 (22.12, 112.39)***	−0.304 (−45.44)	0.38 (−44.76, 45.51)	N.S.
Palpitations mm	Men	4.21 ± 7.93	4.00 ± 9.32	1.50 ± 3.48	0.29 ± 1.07	Peak	3.93 (−1.85, 9.70)	1.21 (−4.56, 7.00)	−0.21 (−5.99, 5.56)	N.S.
		7.43 ± 20.48	3.80 ± 8.18	2.21 ± 5.09	0.21 ± 0.80	AUC_0–8 h_	7.21 (−2.11, 16.54)	2.00 (−7.33, 11.33)	−3.63 (−12.95, 5.70)	N.S.
	Women	1.07 ± 2.13	3.43 ± 5.27	2.36 ± 8.54	0.79 ± 2.94	Peak	0.29 (−5.50, 6.60)	1.57 (−4.20, 7.35)	2.36 (−3.42, 8.13)	N.S.
		1.11 ± 2.18	4.46 ± 7.37	2.62 ± 9.68	1.21 ± 4.54	AUC_0–8 h_	−0.11 (−9.43, 9.22)	1.41 (−7.92, 10.74)	3.36 (−5.97, 12.68)	N.S.
Anxiety mm	Men	0.21 ± 0.80	0.43 ± 1.09	0.21 ± 0.80	0.00 ± 0.00	Peak	0.21 (−3.01, 3.44)	0.21 (−3.01, 3.44)	0.21 (−3.01, 3.44)	N.S.
		0.18 ± 0.67	0.27 ± 0.59	0.11 ± 0.40	0.00 ± 0.00	AUC_0–8 h_	0.18 (−8.34, 8.70)	0.11 (−8.41, 8.63)	0.09 (−8.43, 8.61)	N.S.
	Women	0.14 ± 0.36	0.43 ± 1.60	2.29 ± 8.27	0.00 ± 0.00	Peak	0.14 (−3.08, 3.44)	−1.86 (−5.08, 1.37)	0.29 (−2.94, 3.51)	N.S.
		0.11 ± 0.29	1.30 ± 4.88	5.95 ± 22.11	0.00 ± 0.00	AUC_0–8 h_	0.11 (−8.41, 8.63)	5.95 (−2.57, 14.64)	1.20 (−7.32, 9.71)	N.S.
Headache mm	Men	17.50 ± 16.22	6.00 ± 10.61	0.50 ± 1.87	0.43 ± 1.34	Peak	17.07 (6.31, 27.83)***	0.07 (−10.69, 10.83)	−11.50 (−22.26, −0.74)*	N.S.
		35.77 ± 42.50	9.20 ± 13.42	0.50 ± 1.87	0.43 ± 1.24	AUC_0–8 h_	35.34 (11.52, 59.16)***	0.07 (−23.75, 23.89)	−26.57 (−50.39, −2.76)*	N.S.
	Women	17.57 ± 20.09	3.07 ± 5.65	0.86 ± 2.68	1.29 ± 4.53	Peak	16.29 (5.52, 27.05)***	−0.43 (−11.19, 10.33)	−14.50 (−25.26, −3.74)**	*
		33.91 ± 44.80	3.00 ± 5.69	0.43 ± 1.34	3.05 ± 10.86	AUC_0–8 h_	30.86 (7.04, 54.67)**	−2.63 (−26.44, 21.19)	−30.91 (−54.73, −7.09)**	*
EAVc1 mm	Men	40.93 ± 29.03	49.00 ± 24.26	1.07 ± 4.01	1.00 ± 3.74	Peak	39.93 (21.54, 58.32)***	0.07 (−18.32, 18.46)	8.07 (−10.32, 26.46)	N.S.
		121.21 ± 88.96	155.29 ± 97.00	5.68 ± 21.25	2.80 ± 10.49	AUC_0–8 h_	118.41 (49.70, 187.12)***	2.875 (−65.83, 71.58)	34.07 (−34.64, 102.78)	N.S.
	Women	50.79 ± 26.10	39.63 ± 22.45	0.07 ± 0.27	1.57 ± 5.88	Peak	49.21 (30.83, 67.60)***	−1.50 (−19.89, to 16.89)	−11.16 (−29.55, 7.23)	N.S.
		180.55 ± 104.73	133.56 ± 85.93	0.14 ± 0.54	3.14 ± 11.76	AUC_0–8 h_	177.41 (108.80, 246.12)***	−3.00 (−71.71, 65.71)	−47.00 (−115.71, 21.71)	N.S.
EAVc2 mm	Men	−50.29 ± 38.87	−59.14 ± 35.80	0.07 ± 0.27	0.00 ± 0.00	Peak	−50.29 (−72.88, −27.69)***	0.07 (−22.52, 22.67)	−8.86 (−31.45, 13.74)	N.S.
		−164.48 ± 150.58	193.48 ± 187.43	0.52 ± 1.94	0.00 ± 0.00	AUC_0–8 h_	−164.48 (−266.41 to −62.55)***	0.52 (−101.41, 102.45)	−29.00 (−130.93, 72.93)	N.S.
	Women	−63.07 ± 32.48	−56.14 ± 31.26	−0.14 ± 0.36	−3.07 ± 11.21	Peak	−60.00 (−82.59, −37.41)***	2.93 (−16.67, 25.52)	6.93 (−15.67, 29.52)	N.S.
		−243.77 ± 150.49	−225.91 ± 141.54	−0.36 ± 0.93	−6.52 ± 22.38	AUC_0–8 h_	−237.25 (−339.18, −135.32)***	6.16 (−95.77 to 108.09	17.86 (−84.07, 119.79)	N.S.
EAVc3 mm	Men	−50.93 ± 35.72	−60.43 ± 34.66	0.00 ± 0.00	0.00 ± 0.00	Peak	−50.93 (−72.32, −29.54)***	−0.00 (−21.39–21.89)	−9.50 (−30.89, 11.89)	N.S.
		−163.86 ± 139.86	−191.43 ± 169.58	0.00 ± 0.00	0.00 ± 0.00	AUC_0–8 h_	−163.86 (−257.39, −70.33)***	−0.00 (−93.53, 93.53)	−27.57 (−121.10, 65.96)	N.S.
	Women	−67.21 ± 31.43	−67.29 ± 25.05	−0.07 ± 0.27	−5.00 ± 18.42	Peak	−62.21 (−83.60, −40.83)	4.93 (−16.46, 26.32)	−0.07 (−21.46, 21.32)	N.S.
		−255.21 ± 145.18	−259.36 ± 136.22	−0.14 ± 0.53	−10.36 ± 36.79	AUC_0–8 h_	−244.86 (−338.39, −151.33)***	10.21 (−83.32, 103.95)	−4.14 (−97.67, 89.39)	N.S.
EAVc4 mm	Men	−62.43±-36.20	−73.64 ± 27.96	−2.50 ± 6.36	−1.50 ± 5.61	Peak	60.93 (−81.92, −39.94)***	−1.00 (−21.99, 19.99)	−11.21 (−32.20, 9.77)	N.S.
		−242.21 ± 183.44	−271.91 ± 177.01	−12.07 ± 30.73	−3.48 ± 13.03	AUC_0–8 h_	−238.73 (−347.27, −130.20)***	−8.59 (−117.12, 99.95)	−29.70 (−138.23, 78.84)	N.S.
	Women	−75.21 ± 28.12	−76.71 ± 26.48	−1.64 ± 5.87	−7.21 ± 26.71	Peak	−68.00 (−88.99, −47.01)***	5.57 (−15.42, 26.56)	−1.50 (−22.49, 19.49)	N.S.
		−331.96 ± 178.96	−327.61 ± 163.53	−3.21 ± 11.74	−14.80±-53.34	AUC_0–8 h_	−317.16 (−425.70, −208.63)***	11.59 (−93.95, 120.124)	4.36 (−104.18, 112.89)	N.S.
BAES-A mm	Men	22.71 ± 13.02	27.21 ± 17.56	7.71 ± 9.32	2.21 ± 5.52	Peak	20.50 (8.06, 32.95)***	5.50 (−6.95, 17.95)	4.50 (−7.95, 16.95)	N.S.
		50.96 ± 44.93	80.84 ± 73.44	25.00 ± 42.67	7.66 ± 22.16	AUC_0–8 h_	43.30 (−4.79, 91.40)	17.34 (−30.76, 65.44)	29.88 (−18.22, 77.97)	N.S.
	Women	25.07 ± 14.07	27.79 ± 15.08	10.86 ± 16.27	4.93 ± 8.48	Peak	20.14 (7.70, 32.60)***	5.93 (−6.52, 18.37)	2.71 (−9.73, 15.16)	N.S.
		64.16 ± 56.61	69.57 ± 54.42	39.07 ± 64.50	13.38 ± 25.60	AUC_0–8 h_	50.79 (2.69, 98.88)	25.70 (−22.40, 73.79)	5.41 (−42.69, 52.51)	N.S.
Dose-adjusted BAES-A mm/g	Men	0.33 ± 0.19	0.39 ± 0.25	0.11 ± 0.13	0.03 ± 0.08	Peak	0.29 (0.08, 0.51)**	0.08 (−0.13, 0.29)	0.07 (−0.15, 0.28)	N.S.
		0.73 ± 0.64	1.16 ± 1.05	0.36 ± 0.61	0.11 ± 0.32	AUC_0–8 h_	0.62 (−0.19, 1.43)	0.25 (−0.56, 1.06)	0.43 (−0.38, 1.24)	N.S.
	Women	0.45 ± 0.26	0.50 ± 0.28	0.20 ± 0.30	0.09 ± 0.15	Peak	0.37 (0.15, 0.58)***	0.11 (−0.10, 0.32)	0.05 (−0.16, 0.26)	N.S.
		1.17 ± 1.03	1.26 ± 0.99	0.71 ± 1.17	0.24 ± 0.47	AUC_0–8 h_	0.92 (0.12, 1.73)*	0.47 (−0.34, 1.28)	−0.10 (−0.71, 0.91)	N.S.
BAES-S mm	Men	18.21 ± 14.71	18.07 ± 16.23	2.57 ± 6.20	1.93 ± 3.52	Peak	16.29 (5.80, 26.77)***	0.64 (−9.84, 11.30)	−0.14 (−10.63, 10.34)	N.S.
		64.55 ± 53.92	67.20 ± 68.65	10.93 ± 28.97	6.71 ± 15.14	AUC_0–8 h_	57.84 (12.90, 102.78)**	4.21 (−40.73, 49.15)	2.64 (−42.30, 47.58)	N.S.
	Women	19.50 ± 16.05	16.00 ± 15.45	1.14 ± 2.88	2.57 ± 4.82	Peak	16.93 (6.44, 27.42)***	−1.43 (−11.92, 9.06)	−3.50 (−13.99, 6.99)	N.S.
		83.59 ± 85.94	55.43 ± 57.62	5.66 ± 17.51	9.68 ± 24.50	AUC_0–8 h_	73.91 (28.97, 118.85)***	−4.02 (−48.96, 40.92)	−28.16 (−73.10, 16.78)	N.S.
Dose-adjusted BAES-S mm/g	Men	0.26 ± 0.21	0.26 ± 0.23	0.04 ± 0.08	0.03 ± 0.05	Peak	0.23 (0.06, 0.40)*	−0.01 (−0.17, 0.17)	−0.00 (−0.17, 0.17)	N.S.
		0.92 ± 0.77	0.96 ± 0.98	0.16 ± 0.41	0.10 ± 0.22	AUC_0–8 h_	0.83 (0.08, 1.57)**	0.06 (−0.69, 0.81)	0.04 (−0.71, 0.78)	N.S.
	Women	0.36 ± 0.29	0.29 ± 0.28	0.02 ± 0.05	0.05 ± 0.09	Peak	0.31 (0.14, 0.48)***	−0.03 (−0.20, 0.15)	−0.06 (−0.24, 0.11)	N.S.
		1.52 ± 1.56	1.01 ± 1.05	0.10 ± 0.32	0.18 ± 0.45	AUC_0–8 h_	1.34 (0.60, 2.09)	−0.07 (−0.82, 0.67)	−0.52 (−1.26, 0.24)	N.S.
ARCI-PCAG Score	Men	3.79 ± 3.70	3.00 ± 3.46	−0.43 ± 2.65	0.64 ± 2.24	Peak	3.14 (0.16, 6.13)*	−1.07 (−4.06, 1.92)	−0.79 (−3.77, 2.20)	N.S.
		13.11 ± 11.70	7.71 ± 10.16	−1.65 ± 6.55	2.15 ± 4.01	AUC_0–8 h_	10.96 (0.03, 21.88)*	−3.81 (−14.73, 7.12)	−5.40 (−16.33, 5.52)	N.S.
	Women	5.07 ± 3.32	3.86 ± 3.59	−0.64 ± 1.78	1.21 ± 2.26	Peak	3.86 (0.87, 6.84)**	−1.86 (−4.84, 1.13)	−1.21 (−4.20, 1.77)	N.S.
		24.77 ± 16.21	16.07 ± 16.40	−2.75 ± 5.51	4.73 ± 8.32	AUC_0–8 h_	20.04 (9.11, 30.96)***	−7.48 (−18.41, 3.44)	−8.70 (−19.62, 2.23)	N.S.
ARCI-MBG score	Men	5.79 ± 3.96	6.36 ± 4.55	2.14 ± 3.32	1.07 ± 2.64	Peak	4.71 (2.41, 7.02)***	1.07 (−1.23, 3.38)	0.57 (−1.73, 2.88)	N.S.
		12.96 ± 12.38	18.88 ± 21.32	7.05 ± 13.96	4.99 ± 14.26	AUC_0–8 h_	7.97 (0.50, 14.43)*	2.06 (−5.41, 9.52)	5.93 (−1.54, 13.40)	N.S.
	Women	6.71 ± 3.95	6.43 ± 4.03	0.93 ± 1.27	0.57 ± 1.16	Peak	6.14 (3.84, 8.45)***	0.36 (−1.95, 2.66)	−0.29 (−2.59, 2.02)	N.S.
		16.46 ± 14.18	17.47 ± 11.04	2.62 ± 4.52	1.50 ± 2.72	AUC_0–8 h_	14.97 (7.50, 22.43)***	1.12 (−6.35, 8.58)	1.01 (−6.46, 8.48)	N.S.
ARCI-LSD score	Men	−0.14 ± 2.18	0.14 ± 2.21	−0.07 ± 1.44	−0.21 ± 1.19	Peak	0.07 (−1.64, 1.79)	0.14 (−1.57, 1.86)	0.29 (−1.43, 2.00)	N.S.
		−1.58 ± 7.74	−1.02 ± 8.44	−1.30 ± 4.55	−0.12 ± 3.27	AUC_0–8 h_	−1.47 (−7.65, 4.72)	1.19 (−7.37, 5.00)	0.56 (−5.62, 6.75)	N.S.
	Women	0.79 ± 2.26	0.79 ± 2.29	0.00 ± 0.88	−0.36 ± 0.63	Peak	1.14 (−0.57, 2.86)	0.36 (−1.36, 2.07)	0.00 (−1.71, 1.71)	N.S.
		3.45 ± 9.75	3.17 ± 8.32	−6.69 ± 2.86	−1.61 ± 2.17	AUC_0–8 h_	5.05 (−1.13, 11.24)	0.92 (−5.26, 7.10)	−0.27 (−6.46, 5.91)	N.S.
ARCI-BG score	Men	1.50 ± 2.77	1.43 ± 2.71	2.57 ± 2.77	1.14 ± 2.03	Peak	0.36 (−1.70, 2.41)	1.43 (−0.62, 3.48)	−0.07 (−2.12, 1.98)	N.S.
		−0.16 ± 8.47	2.12 ± 9.78	6.62 ± 7.40	3.84 ± 9.01	AUC_0–8 h_	−4.00 (−10.62, 2.62)	2.78 (−3.84, 9.40)	2.28 (−4.34, 8.90)	N.S.
	Women	0.50 ± 3.13	2.43 ± 2.17	1.36 ± 1.86	0.86 ± 1.61	Peak	−0.36 (−2.41, 1.70)	0.50 (−1.55, 2.55)	1.93 (−0.12, 3.98)	N.S.
		−1.09 ± 9.62	2.75 ± 9.28	4.84 ± 6.41	2.35 ± 4.85	AUC_0–8 h_	−3.44 (−10.06, 3.18)	2.49 (−4.13, 9.11)	3.84 (−2.78, 10.46)	N.S.
ARCI-A score	Men	3.86 ± 1.56	3.43 ± 1.83	2.29 ± 2.43	1.43 ± 2.65	Peak	2.43 (0.72, 4.13)**	0.86 (−8.85, 5.56)	−0.43 (−2.13, 1.28)	N.S.
		9.21 ± 6.82	11.39 ± 11.06	7.57 ± 9.61	6.13 ± 13.52	AUC_0–8 h_	3.09 (−2.39, 8.57)	1.45 (−4.03, 6.93)	2.18 (−3.30, 7.66)	N.S.
	Women	3.79 ± 2.01	3.86 ± 1.96	1.50 ± 1.74	0.86 ± 1.41	Peak	2.93 (1.22, 4.63)***	0.64 (−1.06, 2.45)	0.07 (−1.63, 1.78)	N.S.
		12.65 ± 9.81	12.79 ± 8.29	5.49 ± 6.27	3.22 ± 4.73	AUC_0–8 h_	9.43 (3.95, 14.91)***	2.27 (−3.21, 7.75)	0.14 (−5.35, 5.62)	N.S.
Like the drug mm	Men	47.00 ± 22.80	56.50 ± 27.00	18.21 ± 26.60	0.93 ± 3.20		46.07 (24.32, 67.82)***	17.29 (−4.47, 39.04)	9.50 (−12.25, 31.25)	N.S.
	Women	56.29 ± 26.30	71.21 ± 18.30	10.07 ± 17.30	7.29 ± 19.00		49.00 (27.25, 70.75)***	2.79 (−18.97, 24.54)	14.93 (−6.82, 36.68)	N.S.
Desire to continue drinking mm	Men	52.77 ± 29.44	55.00 ± 30.01	16.36 ± 22.20	8.57 ± 15.49		43.82 (14.00, 73.65)***	7.79 (−21.41, 36.98)	2.61 (−2.77, 55.63)	N.S.
	Women	52.0 ± 32.33	49.57 ± 30.00	22.93 ± 35.00	23.14 ± 39.64		28.86 (−0.34, 58.06)	−0.21 (−29.41, 28.98)	−2.43 (−31.63, 26.77)	N.S.

n = 14 men in all conditions and n = 14 women in all conditions. A, alcohol + placebo energy drink; A/ED, alcohol + energy drink; ARCI, Addiction Research Center Inventory; ARCI-PCAG, pentobarbital–chlorpromazine–alcohol group; ARCI-MBG, morphine–benzedrine group; ARCI-LSD, lysergic and diethylamide scale; ARCI-BG, benzedrine group; ARCI-A, amphetamine; AUC_0-8 h_, area under the curve of effects from 0 until 8 h; BAES-A, Biphasic Alcohol Effects Scale activation/stimulation; BAES-S, Biphasic Alcohol Effects Scale sedation; CI, confidence interval; EAVc1, driving capability; EAVc2, willingness to drive an ill child to the hospital; EAVc3, willingness to drive a sick friend home; EAVc4, willingness to drive a friend to a party; ED, energy drink + placebo alcohol; ES, effect size; P, placebo alcohol + placebo energy drink; SD, standard deviation; N.S., not significant; *p < 0.05; **p < 0.01; ***p < 0.001.

**TABLE 4:** Results of vital signs.

Outcome	Gender	A Mean ± SD	A/ED Mean ± SD	ED Mean ± SD	*p* Mean ± SD	Parameter	ES (95% CI) A	ES (95% CI) ED	ES (95% CI) A/ED vs. A	Interaction
SBP mmHg	Men	−5.25 ± 14.01	9.14 ± 13.73	8.32 ± 10.78	−0.07 ± 12.46	Peak	−5.18 (−17.98, 7.62)	8.39 (−4.41, 21.19)	14.93 (1.60, 27.19)**	N.S.
		−46.49 ± 38.53	8.05 ± 43.75	15.26 ± 34.70	−5.55 ± 45.33	AUC_0–8 h_	−40.94 (−79.41, −2.47)*	20.81 (−17.66, 59.28)	54.55 (16.08, 93.02)**	N.S.
	Women	1.79 ± 15.36	0.54 ± 15.58	5.39 ± 11.20	−5.11 ± 10.72	Peak	6.89 (−5.90, 19.69)	10.50 (−2.30, 23.30)	−1.25 (−14.05, 11.55)	N.S.
		−37.36 ± 43.42	−16.08 ± 52.16	17.42 ± 44.21	−29.33 ± 27.94	AUC_0–8 h_	−8.03 (−46.50, 30.45)	46.75 (8.28, 85.22)**	21.28 (−17.19, 59.75)	N.S.
DBP mmHg	Men	−15.79 ± 5.27	2.89 ± 16.03	−2.25 ± 9.10	−3.50 ± 9.30	Peak	−12.29 (−23.32, −1.25)*	1.25 (−9.78, 12.28)	18.68 (7.65, 29.71)***	***
		−71.77 ± 35.27	−39.17 ± 54.79	−10.85 ± 22.95	−14.57 ± 31.00	AUC_0–8 h_	−57.20 (−90.93, −23.46)***	3.72 (−30.01, 37.46)	32.60 (−1.34, 66.34)	N.S.
	Women	−12.89 ± 9.91	−7.14 ± 13.16	3.75 ± 9.70	−7.61 ± 6.90	Peak	−5.29 (−16.32, 5.75)	11.36 (0.32, 22.40)*	5.75 (−5.28, 16.78)	N.S.
		−54.69 ± 29.58	−38.34 ± 33.22	7.79 ± 31.98	−27.33 ± 25.82	AUC_0–8 h_	−27.36 (−61.09, 3.38)	35.13 (1.39, 68.86)*	16.35 (−17.39, 50.09)	N.S.
Heart rate bpm	Men	23.21 ± 4.91	14.57 ± 12.80	3.43 ± 12.34	9.18 ± 9.06	Peak	14.04 (4.38, 23.70)**	−5.75 (−15.41, 3.91)	−8.64 (−18.30, 1.02)	N.S.
		113.20 ± 32.21	68.29 ± 58.71	2.29 ± 39.92	38.31 ± 32.79	AUC_0–8 h_	74.88 (38.18, 111.59)***	−36.03 (−72.73, 0.68)	−44.91 (−81.62, −8.21)**	N.S.
	Women	20.07 ± 8.91	13.18 ± 11.67	2.82 ± 12.15	3.68 ± 9.33	Peak	16.39 (6.73, 26.05)***	−0.86 (−10.51, 29.45)	−6.89 (−16.55, 2.77)*	N.S.
		86.89 ± 52.36	46.54 ± 41.85	13.87 ± 41.62	10.26 ± 26.70	AUC_0–8 h_	76.63 (39.93, 113.34)***	3.61 (−33.01, 40.31)	−40.35 (−77.06, −3.64)*	*
Temperature °C	Men	−0.27 ± 0.23	0.10 ± 0.43	−0.05 ± 0.53	−0.09 ± 0.39	Peak	−0.19 (−0.59, 0.22)	0.04 (−0.37, 0.44)	0.38 (−0.03, 0.78)	N.S.
		−0.60 ± 1.00	0.35 ± 1.31	0.27 ± 1.62	0.09 ± 1.03	AUC_0–8 h_	−0.70 (−2.07, 0.67)	0.18 (−1.19, 1.55)	0.95 (−0.42, 2.32)	N.S.
	Women	−0.03 ± 0.40	−0.21 ± 0.45	−0.16 ± 0.49	−0.04 ± 0.37	Peak	0.01 (−0.40, 0.41)	−0.13 (−0.53, 0.28)	0.19 (−0.59, 0.22)	N.S.
		−0.22 ± 1.15	−0.54 ± 1.61	0.19 ± 1.77	−0.03 ± 1.00	AUC_0–8 h_	−0.20 (−1.56, 1.17)	0.22 (−1.15, 1.60)	−0.32 (1.69, 1.05)	N.S.
Urine 0–8 h mL	Men	1831 ± 288	2,311 ± 452	2022 ± 375	1826 ± 242		4.86 (−267.33, 277.04)	196.43 (−75.76, 468.612)	479.79 (207.60, 751.97)***	N.S.
	Women	1791 ± 287	2002 ± 318	1941 ± 308	1810 ± 289		−18.21 (−290.40, 253.97)	131.07 (−141.11, 403.26)	210.72 (−61.47, 482.90)	N.S

n = 14 men in all conditions and n = 14 women in all conditions. A, alcohol + placebo energy drink; A/ED, alcohol + energy drink; AUC_0–8 h_, area under the curve of effects from 0 until 8 h; CI, confidence interval; DBP, diastolic blood pressure; ED, energy drink + placebo alcohol; ES, effect size; P, placebo alcohol + placebo energy drink; SBP, systolic blood pressure; SD, standard deviation. N.S., not significant; *p < 0.05; **p < 0.01; ***p < 0.001.

At equivalent doses (same alcohol and caffeine concentrations), gender differences were detected only in drunkenness and sedation (ARCI-PCAG). Effect sizes between men and women, which were statistically significant, and trends are reported in Table 5.

**TABLE 5:** Summary of significant effects in differences between men and women among different treatment conditions.

Outcome	Parameter	A ES (95% CI)	A/ED ES (95% CI)	ED ES (95% CI)	P ES (95% CI)
Errors (TT)	Peak	−6.50 (−13.23, 0.23)ˆ	N.S.	N.S.	N.S.
Dose-adjusted mean latency ms/g (PVT)	Peak	N.S.	0.40 (−0.03, 0.84)ˆ	N.S.	N.S.
	AUC_0–6 h_	1.48 (−0.07, 3.03)ˆ	N.S.	N.S.	N.S.
Drunkenness mm	AUC_0–8 h_	44.82 (5.07, 84.57)*	N.S.	N.S.	N.S.
Dose-adjusted drunkenness mm/g	Peak	0.26 (0.04, 0.49)*	N.S.	N.S.	N.S.
	AUC_0–8 h_	1.11 (0.43, 1.79)***	0.65 (−0.03, 1.33)ˆ	N.S.	N.S.
Drowsiness mm	AUC_0–8 h_	58.84 (−6.70, 124.36)ˆ	N.S.	N.S.	N.S.
Dizziness mm	AUC_0–8 h_	N.S.	42.95 (−3.01, 88.90)ˆ	N.S.	N.S.
EAVc1 mm	AUC_0–8 h_	59.34 (−4.11, 122.79)ˆ	N.S.	N.S.	N.S.
EAVc3 mm	AUC_0–8 h_	−91.36 (−190.16, 7.45)ˆ	N.S.	N.S.	N.S.
ARCI-PCAG Score	AUC_0–8 h_	11.61 (1.51, 21.81)*	N.S.	N.S.	N.S.
DBP mmHg	Peak	N.S.	−10.04 (−19.84, −0.24)*	N.S.	N.S.
Urine 8 h volume mL		N.S.	−308.57 (−611.76, −5.38)*	N.S.	N.S.

Reported outcomes are those with statistically significant differences or a trend, among both genders. n = 14 men in all conditions (except n = 13 in placebo for PVT and TT outcomes) and n = 14 women in all conditions. A, alcohol + placebo energy drink; A/ED, alcohol + energy drink; ARCI, Addiction Research Center Inventory; ARCI-PCAG, pentobarbital–chlorpromazine–alcohol group; AUC, area under the curve of effects CI, confidence interval; DBP, diastolic blood pressure; EAVc1, driving capability; EAVc3, willingness to drive a sick friend home; ED, energy drink + placebo alcohol; ES, effect size; P, placebo alcohol + placebo energy drink; PVT, psychomotor vigilance task; TT, tracking test; N.S., not significant; *p < 0.05; **p < 0.01; ***p < 0.001. ˆ p < 0.1.

#### 3.3.1 Driving-related skills

**FIGURE 3:**
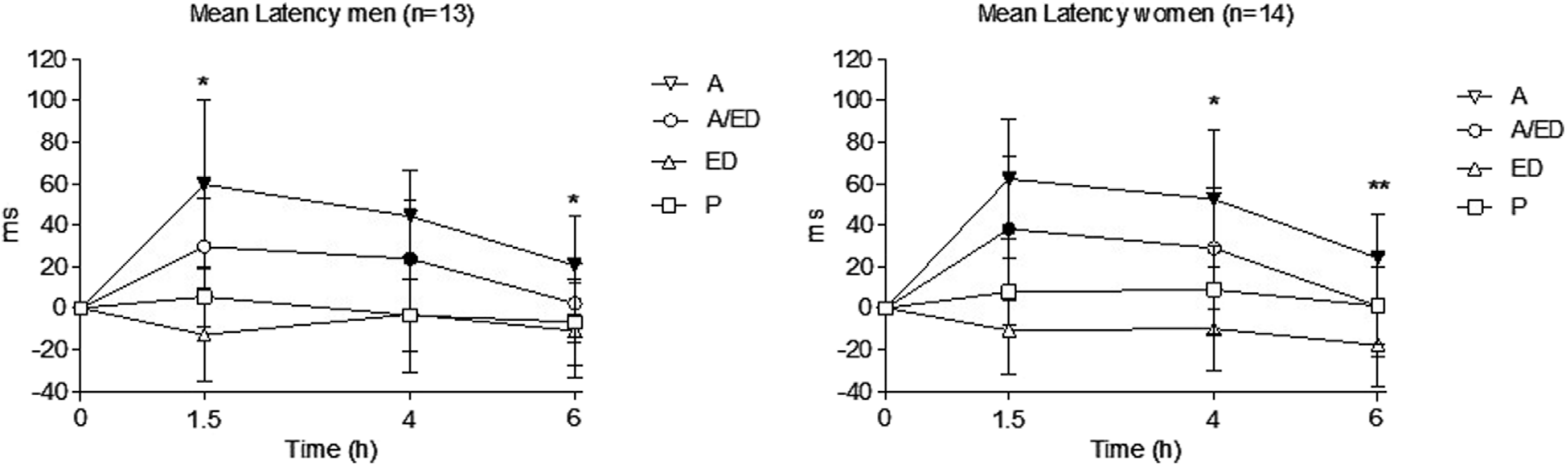
Mean latency in the PVT. Data points and error bars represent the mean and SD values. A, alcohol + energy drink placebo; A/ED, alcohol + energy drink; ED, energy drink + placebo alcohol; P, placebo alcohol + placebo energy drink. *p < 0.05; **p < 0.01. Filled symbols indicate a significant difference from placebo (p < 0.05). Significance is only reported for the comparison of primary interest (A vs. A/ED) and between all conditions and placebo. Differences were analyzed using a repeated measures ANOVA, with treatment and time as factors.

Data from one man on placebo were excluded due to outlier results (based on the Dixon test). Effects along 6 h in mean latency (PVT) are shown in Figure 3, while time spent outside the road is shown in Supplementary Figure S1 (TT). In both alcoholic conditions, mean latency and time spent outside the road increased, peaking mainly at 1.5 h, and impairment progressively decreased until 6 h. Less impairment was observed with the addition of EDs to alcohol, reaching statistical significance at several time points in men and women (see Supplementary Table S1 and both figures).

No gender differences in peak or AUC were observed in mean latency, median latency, and SD latency in any of the treatment conditions. A trend toward greater impairment in women than in men with alcohol was observed in mean latency but only in the dose-adjusted results.

EDs reduced mean latency compared to placebo, with the reduction reaching statistical significance in women (peak). The A/ED condition, compared to the A condition, caused a statistically significant decrease in peak mean latency in men. This reduction was also observed in both genders in the AUC of effects. For peak median latency, A/ED values were lower than those for alcohol alone in women, while AUC values were again reduced with the addition of EDs in both genders.

No gender differences were found for the time spent outside the road and the number of gyres. In the peak number of errors, a trend for higher values in men was observed with alcohol. The A/ED condition, compared with the A condition, reduced the number of errors and time spent outside the road, but these differences were statistically significant for peak and AUC values only in men. Furthermore, the number of errors at 1.5 h was also lower with A/ED in men (see Supplementary Table S1).

An alcohol effect was shown in the Maddox Wing, but no gender differences or differences in A/ED vs. A conditions were found.

#### 3.3.2 Subjective effects

**FIGURE 4:**
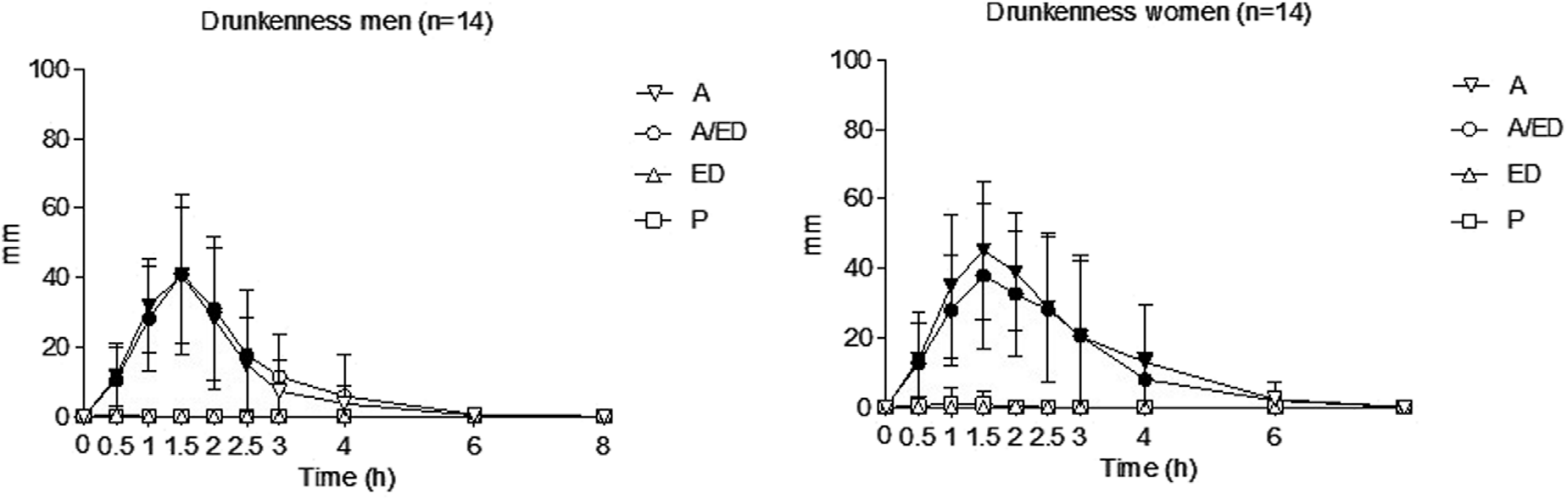
Drunkenness. Data points and error bars represent the mean and SD values. A, alcohol + energy drink placebo; A/ED, alcohol + energy drink; ED, energy drink + placebo alcohol; P, placebo alcohol + placebo energy drink. *p < 0.05; **p < 0.01. Filled symbols indicate a significant difference from placebo (p < 0.05). Significance is only reported for the comparison of primary interest (A vs. A/ED) and between all conditions and placebo. Differences were analyzed using a repeated measures ANOVA, with treatment and time as factors.

Moderate drunkenness was observed in both genders, with peak values at 1.5 h and disappearing at 6 h (see Figure 4). There were no differences between the A/ED vs. A conditions in any of the genders. In the alcohol condition, drunkenness was higher in women than in men (AUC and dose-adjusted peak/AUC).

Drowsiness and dizziness were reported with the A and A/ED conditions. A trend was observed indicating more drowsiness (A condition, AUC) and dizziness (A/ED condition, AUC) in women than in men. No statistically significant differences were found between the A/ED and A conditions.

Heart palpitations were not reported by most of the participants, while there were no scores in anxiety. Mild headache was reported mainly at 6–8 h after administration, clearly lower in the A/ED condition compared to the A condition in both genders (peak/AUC) and also at 8 h in women. None of the participants reported habitual headaches during the medical interview.

At the end of the sessions, participants reported greater liking for the combination of A/ED than A, but this difference was not statistically significant. Both alcoholic conditions produced a desire to continue drinking. Mild activation and sedation were detected using the BAES, with no statistically significant differences between genders or between the A/ED and A conditions, even after adjusting for the administered dose.

A 50% reduction in subjective driving capability, was observed at 1.5 h in both alcoholic conditions, with a return to baseline at 6–8 h. Moreover, an alcohol effect was found in willingness to drive under certain circumstances (it was reduced in all of them), but no differences were found between the A and A/ED conditions in these outcomes. In the alcohol condition, women tended to report more impairment in subjective driving capability (AUC) and expressed less willingness to drive a sick friend home (AUC) than men.

**FIGURE 5:**
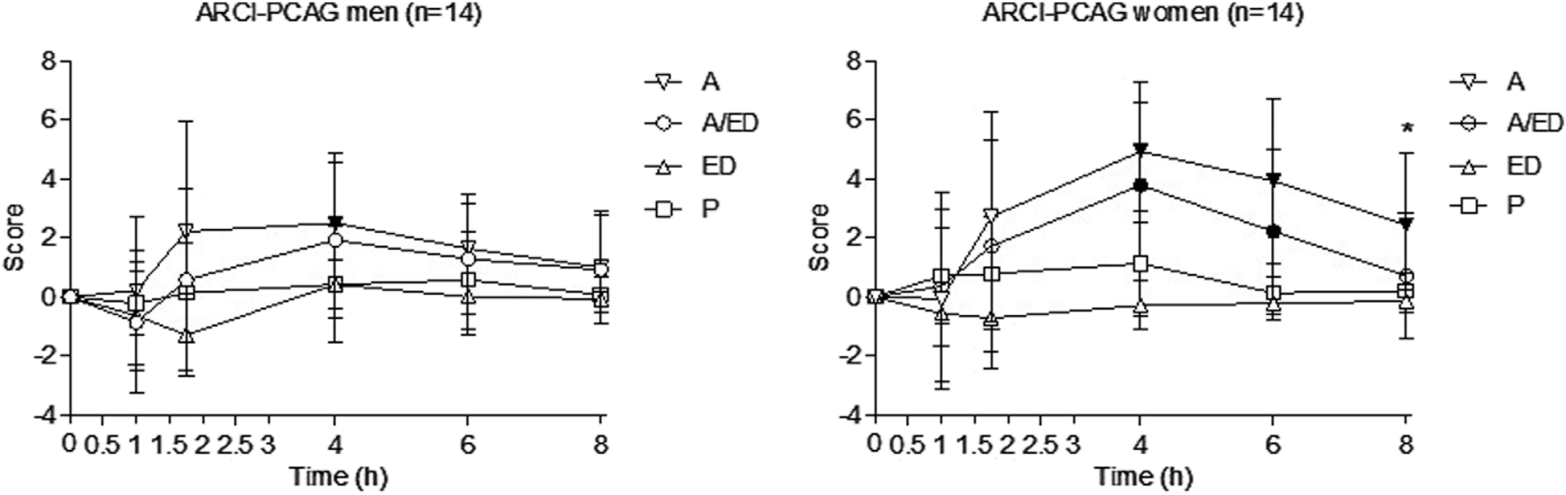
Sedation (ARCI-PCAG). Data points and error bars represent the mean and SD values. A, alcohol + energy drink placebo; A/ED, alcohol + energy drink; ED, energy drink + placebo alcohol; P, placebo alcohol + placebo energy drink. *p < 0.05; **p < 0.01. Filled symbols indicate a significant difference from placebo (p < 0.05). Significance is only reported for the comparison of primary interest (A vs. A/ED) and between all conditions and placebo. Differences were analyzed using a repeated measures ANOVA, with treatment and time as factors.

Subjects reported sedation, as measured by the ARCI-PCAG subscale, under both alcoholic conditions, with the effect being most pronounced at 4 h; in contrast, negative sedation values were observed in the ED condition at 1.75 h. Women reported higher sedation than men in the alcohol condition (AUC and at 4 and 6 h). Although sedation was higher in the A condition than in the A/ED condition, this difference did not reach statistical significance for peak or AUC values, except at 8 h in the case of women (see Supplementary Table S1; Figure 5).

Euphoria, as measured by the ARCI-MBG subscale, was observed with both alcoholic conditions with peak values at 1 h. Energy and intellectual efficiency (ARCI-BG) were very mild in all conditions. An increased sense of wellbeing (ARCI-A) was observed with both alcoholic conditions, with peak values at 1 h. Dysphoria (ARCI-LSD) was not reported. No gender differences were found across these subscales.

#### 3.3.3 Vital signs

Both conditions with alcohol increased the heart rate from 0.5 to 8 h, with peak effects of 15–20 bpm occurring at 2.5–2.75 h. In both genders, a lower increase was observed with the A/ED condition compared to the A condition. No gender differences were found.

No alcohol-related effects were found on systolic blood pressure, while EDs slightly increased it in women (AUC). A difference favoring higher systolic blood pressure with A/ED compared to A was found in men (peak, AUC). No gender differences were found.

In turn, alcohol reduced diastolic blood pressure in both genders, with the reduction reaching statistical significance only in men (peak, AUC). A difference favoring a small reduction in diastolic blood pressure with A/ED compared to alcohol was found in men. Regarding gender comparisons in the A/ED condition, a negative peak value was observed only in women.

Women had lower peak diastolic blood pressure than men in the A/ED condition. Again, a difference between the A and A/ED conditions, with lower pressure with alcohol alone, was found in men (peak and at 8 h) (see Supplementary Table S1).

Finally, there were no significant effects on the oral temperature. In men, the amount of urine excreted was higher with the A/ED condition than with the other conditions. The amount of urine collected was also higher in men than in women with the A/ED condition.

### 3.4 Adverse events

All beverages were well tolerated, and no participants experienced vomiting. Only mild adverse events were reported by three volunteers (headache, a hematoma at the puncture site, and a knee contusion). The first was considered related to treatment, the second was associated with the procedures of the study, and the third was non-related.

### 3.5 Correlations

There was a strong and positive correlation between BrAC and drunkenness feeling, capability to drive, BAES-A, ARCI-A, and ARCI-MBG in both conditions with alcohol and in both genders. The correlation was moderate for dizziness. In the case of driving-related skills, a strong correlation was observed in mean latency, time spent outside the road, and number of errors, with stronger associations in the A condition than in the A/ED condition. Measures of sedation like BAES-S showed a moderate correlation with alcohol concentrations, while drowsiness had a weak correlation in the A condition, and ARCI-PCAG showed no correlation at all (see Table 6).

**TABLE 6:** Summary of correlations between breath alcohol concentrations and effects between alcohol and A/ED.

Outcome	Gender	A r ± 95% CI	A/ED r ± 95% CI
Drunkenness	Men	0.69 (0.58, 0.78)	0.70 (0.59, 0.78)
	Women	0.77 (0.68, 0.83)	0.73 (0.63, 0.80)
Drowsiness	Men	0.22 (0.03, 0.39)	0.02 (−0.17, 0.21)
	Women	0.21 (0.03, 0.38)	0.35 (0.18, 0.51)
Dizziness	Men	0.44 (0.27, 0.58)	0.40 (0.23, 0.55)
	Women	0.56 (0.42, 0.68)	0.56 (0.41, 0.67)
EVAc1	Men	0.77 (0.6, 0.87)	0.82 (0.68, 0.90)
	Women	0.83 (0.70, 0.91)	0.79 (0.64, 0.89)
Mean latency (PVT)	Men	0.64 (0.35, 0.82)	0.59 (0.27, 0.80)
	Women	0.62 (0.31, 0.81)	0.56 (0.23, 0.78)
Time out (TT)	Men	0.66 (0.37, 0.83)	0.44 (0.08, 0.71)
	Women	0.73 (0.48, 0.87)	0.58 (0.26, 0.79)
Errors (TT)	Men	0.72 (0.48, 0.87)	0.39 (0.01, 0.67)
	Women	0.71 (0.44, 0.86)	0.75 (0.51, 0.88)
BAES-A	Men	0.65 (0.50, 0.76)	0.66 (0.52, 0.77)
	Women	0.63 (0.47, 0.74)	0.61 (0.45, 0.73)
BAES-S	Men	0.48 (0.29, 0.63)	0.3 (0.09, 0.49)
	Women	0.30 (0.09, 0.49)	0.33 (0.12, 0.51)
ARCI-A	Men	0.68 (0.51, 0.80)	0.72 (0.56, 0.83)
	Women	0.66 (0.48, 0.79)	0.71 (0.55, 0.82)
ARCI-BG	Men	0.39 (0.13, 0.59)	0.36 (0.11, 0.57)
	Women	0.37 (0.11, 0.58)	0.39 (0.14, 0.60)
ARCI-LSD	Men	0.00 (−0.26, 0.27)	0.21 (−0.06, 0.45)
	Women	0.36 (0.11, 0.57)	0.36 (0.10, 0.57)
ARCI-MBG	Men	0.70 (0.53, 0.81)	0.73 (0.57, 0.83)
	Women	0.65 (0.47, 0.78)	0.68 (0.51, 0.80)
ARCI-PCAG	Men	0.01 (−0.26, 0.27)	−0.27 (−0.50, −0.00)
	Women	−0.14 (−0.39, 0.13)	−0.07 (−0.20, −0.33)

n = 14 men and n = 14 women in all outcomes. A, alcohol + placebo energy drink; A/ED, alcohol + energy drink; ARCI, Addiction Research Center Inventory; ARCI-PCAG, pentobarbital–chlorpromazine–alcohol group; ARCI-MBG, morphine–benzedrine group; ARCI-LSD, lysergic and diethylamide scale; ARCI-BG, benzedrine group; ARCI-A, amphetamine; BAES-A, Biphasic Alcohol Effects Scale activation; BAES-S, Biphasic Alcohol Effects Scale sedation; CI, confidence interval; EAVc1, driving capability; PVT, psychomotor vigilance task; TT, tracking test.

## 4 Discussion

In this study, after administering alcohol and energy drinks in a binge-drinking pattern, greater drunkenness was found in women with alcohol, a trend also observed when mixed with EDs, despite similar breath alcohol concentrations between men and women. Additionally, with alcohol administration, women also reported higher sedation (ARCI-PCAG) than men, reflecting higher sensitivity to alcohol-induced sedation. Nevertheless, we could not demonstrate gender differences in driving-related skills with alcohol or AmEDs, although a trend for higher impairment in reaction time was observed in women than in men.

Furthermore, although EDs slightly reduced the effects of alcohol on several of the outcomes measured, they failed to fully counteract its effects, and no interaction was found between both beverages. This fact is relevant since young people mix alcohol with EDs to become less intoxicated, and the results do not support this practice.

Defining concentrations of binge drinking (0.46 mg/L in breath air, equivalent to more than 80 mg/dL in the blood) were achieved in both genders. Our results are applicable to this type of consumption pattern, and therefore, the experimental design used can be applied to future studies assessing binge-drinking effects. Furthermore, there were gender differences in the alcohol concentrations achieved since women received a dose 21% lower than men, allowing us to compare acute effects without being influenced by gender differences in alcohol pharmacokinetics (increased bioavailability and faster disappearance rates in women) (Mumenthaler et al., 1999; Baraona et al., 2001). When alcohol concentrations were adjusted to the administered dose, as expected, higher alcohol concentrations were observed in women. With the administration of the same dose in both genders, higher concentrations can be expected in women after a binge-drinking episode (Poyatos et al., 2019).

By adding EDs to alcohol, our results detected a reduction in the AUC of alcohol concentrations in breath air (6%), with no differences in peak values. Another study, which also administered high doses of EDs, found lower alcohol concentrations with AmEDs in the ascending limb (Peacock et al., 2015). The authors argued that lower alcohol concentrations were found when mixing alcohol with natural sweeteners, as in the case of EDs, compared to artificial sweeteners (Marczinski and Stamates, 2013; Rossheim and Thombs, 2011; Wu et al., 2006). Red Bull Red Edition contained glucose, saccharose, and fructose, while its placebo (Strawberry Fanta) contained high fructose corn syrup. The total amount of sugar was similar among beverages (11 g/100 mL and 12.4 g/100 mL, respectively). Nevertheless, most of the studies have shown no effect of EDs in BrACs (McKetin et al., 2015). In our opinion, disparities between studies are a consequence of the different doses and ingredients of the EDs administered and their placebos. Nevertheless, it should be noted that the difference found in our study was minimal.

Regarding caffeine, higher concentrations were detected when it was combined with alcohol, suggesting a pharmacokinetic interaction between caffeine and alcohol, as previously described (Pérez-Mañá et al., 2022; Mitchell et al., 1983; George et al., 1986; Azcona et al., 1995; Gazzaz et al., 2018). It is postulated that alcohol inhibits the metabolism of caffeine through CYP1A2, causing lower caffeine clearance and lengthening its elimination half-life. The interaction was present in both genders.

We found that alcohol increased the reaction time in the PVT, the time spent outside the road, and the number of errors in the tracking test in both genders. The results obtained were as expected, given that alcohol has been widely demonstrated to impair psycho-motor performance and driving-related skills (Miller et al., 2009; Mackay et al., 2002; Jongen et al., 2014; Benson et al., 2019).

In our study, ED had a main effect on peak mean reaction time, but only in women, and no interaction between the beverages was found. Similar results were obtained when beer was mixed with caffeine (383 mg in men and 338 mg in women), where caffeine had no main or interacting effects with alcohol on mean reaction time, also measured with PVT (Howland et al., 2011). Previously, a decrease (faster) in mean reaction time in PVT following ED administration was also reported (Antonio et al., 2019). The improvement in reaction time with EDs is a consequence of the enhancing effects of caffeine on attention tasks (Childs and de Wit, 2006; Haskell et al., 2005; Heatherley et al., 2005; Mclellan et al., 2016; Saville et al., 2018).

Mixed results regarding the effects of adding EDs to alcohol on psychomotor performance and driving-related skills have been found. Studies have been carried out with different measuring instruments and different doses, making it more difficult to draw conclusions. Ferreira et al. (2013) did not detect an improvement in motor coordination and visual reaction time (Ferreira et al., 2013). Others reported that EDs reduced alcohol-induced impairment, but a consistent pattern was not found in all tests performed (Marczinski et al., 2017; Alford et al., 2012; Peacock and Bruno, 2015). McKetin et al. (2015) concluded that EDs only improve some aspects of complex tasks (McKetin et al., 2015).

In our case, a reduction in mean reaction time was observed with AmEDs (AUC and at several time-points) compared with A, but EDs did not fully counteract alcohol-related effects. Furthermore, EDs slightly reduced the impairment produced by alcohol in the tracking test. The results are congruent with those previously obtained with 60 g of alcohol and 750 mL of EDs in men (Pérez-Mañá et al., 2022).

However, no gender differences in driving-related skills were found. Only for dose-adjusted mean reaction time, a trend for worse performance in women was observed in the A and A/ED conditions, while a trend for a higher increase in the number of errors was observed in men in the A condition. Other individual studies have also failed to detect gender differences in driving-related skills after alcohol administration, with such differences only becoming apparent when data were aggregated, supporting the notion of higher sensitivity to alcohol-induced impairment in women (Miller et al., 2009).

In our study, greater drunkenness was reported in women than in men when they received alcohol, but we did not observe a reduction in drunkenness with AmEDs. This lack of effect of EDs is widely reported throughout the literature, with both EDs and caffeine (Benson and Scholey, 2014; Alford et al., 2012; Howland et al., 2011; Liguori and Robinson, 2001; Ferreira et al., 2006; Marczinski and Fillmore, 2003; Peacock et al., 2013).

No gender differences were found in activation and sedation measured by the BAES, consistent with the findings from a previous study (Marczinski et al., 2012). Higher activation and less sedation with AmEDs than with alcohol were expected, as observed in our previous study (Pérez-Mañá et al., 2022). These results were not replicated in the current study, which may be due to the differences in the dose of alcohol administered (14% higher in the present study) and the rhythm of administration.

Participants reported a reduced capability to drive and a lower willingness to drive under certain circumstances in both alcoholic conditions compared to those where alcohol was not present. No differences were observed between the A/ED and A conditions, although such differences were found in our previous study (Pérez-Mañá et al., 2022). Moreover, the discrepancies may be related to alcohol administration. No gender differences were found in the perceived ability to drive, similar to the findings of Marczinski et al. (2012). Trends observed in willingness to drive would support a lower predisposition of women to drive intoxicated in certain circumstances.

Desire to keep drinking and like the drug also showed a main effect of alcohol, but no differences were found between the A and A/ED conditions, consistent with our previous study (Pérez-Mañá et al., 2022). Another study, however, supports that AmED beverages lead to a greater desire to drink alcohol compared to the same amount of alcohol consumed alone in a dose-dependent manner (Marczinski et al., 2016).

Although less drowsiness was observed with AmEDs compared to alcohol, this difference was not statistically significant in our study. Previous studies with caffeine have reported an improvement in alcohol-induced drowsiness (Drake et al., 2003; Buela-Casal et al., 1994). Disparities could be explained by different alcohol and caffeine doses. Furthermore, a tendency toward greater drowsiness in the alcohol condition was observed in women, suggesting that they may experience more pronounced negative subjective effects of alcohol than men (greater drunkenness and sedation have been previously mentioned). EDs have not been found to reduce dizziness, as reported in a previous study with caffeine (Drake et al., 2003) and EDs (Pérez-Mañá et al., 2022). Moreover, a trend toward greater dizziness was found in women compared to men in this case with AmEDs.

A reduction in headache, which appeared after 6–8 h, was observed when EDs were added to alcohol, as reported in a previous study (Ferreira et al., 2006). Other studies, however, did not report this effect (Peacock et al., 2014b; Pérez-Mañá et al., 2022). The discrepancies can be explained by the different doses administered and evaluation times.

In both conditions with alcohol, an increase in the heart rate was detected, while a reduction in blood pressure was found along with the experimental sessions. These data are consistent with those of previous studies showing that high doses of alcohol lead to a reduction in blood pressure and an increase in the heart rate in the first 12 h (Tasnim et al., 2020). Increased heart rate can be explained by the activation of the sympathetic nervous system with alcohol (Spaak et al., 2010), while reduced blood pressure is due to a reduction in vasoconstrictors and an increase in vasodilators after alcohol intake (Tasnim et al., 2020). The effects of EDs on blood pressure can be explained by the peripheral vasoconstrictive effects of caffeine (Fletcher et al., 2017). Additionally, another study suggests that ED can reduce the heart rate because of its effects on blood pressure (Oberhoffer et al., 2022).

In addition, a greater volume of urine was generated with AmEDs, which can be explained by the diuretic properties of alcohol and caffeine, which are more pronounced in men, replicating the results of our previous study in men (Pérez-Mañá et al., 2022).

Our study has both strengths and limitations. We administered high doses of both alcohol and EDs, simulating a binge-drinking episode, which is the usual consumption pattern among young people. Therefore, the results can be easily extrapolated to real-world consumption situations. Another strength is the full factorial design, which allows for estimating the effects of each drink separately and their interaction. Finally, unlike most published studies, we focused on analyzing gender differences.

It should be noted that only five volunteers correctly guessed the combination of drinks they had drunk each day. This fact reflects that the drinks were correctly masked, minimizing any possible biases induced by knowledge of the assigned treatment.

The main limitation to our study is the sample size, which may be underpowered to detect modest differences between genders or treatments in secondary outcomes. Additionally, we assessed driving-related skills using a tracking test, and the results obtained are difficult to extrapolate to real-world driving conditions. Future studies should be conducted using a driving simulator to study gender differences in the effects of AmEDs on driving performance. The simulator allows for calculating the weaving of the car, which is measured by the standard deviation of the lateral position.

## 5 Conclusion

The administration of AmEDs in a binge-drinking pattern to healthy volunteers produced the prototypical effects of alcohol, which were not counteracted by energy drinks. Women reported higher levels of drunkenness and sedation than men, but no gender differences were observed in driving-related skills. Further studies should be conducted to elucidate the gender-specific effects of AmEDs on driving performance.

## Data Availability

The raw data supporting the conclusions of this article will be made available by the authors without undue reservation.
