# Supplementary material for: No significant gender differences in driving-related skills following alcohol mixed with energy drinks during an experimental binge-drinking episode
Source: Front Pharmacol. 2025 May 23;16:1581229. doi: 10.3389/fphar.2025.1581229 (PMC12142690; doi:10.3389/fphar.2025.1581229)
Supplement: Supplementary file 1 [file DataSheet1.pdf]

Supplementary Material

1 Supplementary Data

Supplementary Material includes one table, one figure and additional information in the methodology section.

2 Methodology

Inclusion Criteria:

1. Males and females between 18-40 years old, weight between 50 and 100 kg and BMI (BMI=weight/height²) between 20-28 kg/m². Lower or higher BMIs will be allowed, if the researchers considered that do not suppose a risk to the subjects and do not interfere with the objectives of the study.
2. Recreational alcohol consumption in form of occasional binge-drinking (≥1 episode / month) and at least consumption of 1 unit (10 g, "standard" drink - one alcoholic drink equivalent) per day or its equivalent over the whole week [7 units, 70 g)]) and having experienced drunkenness several times
3. Regular consumption of beverages containing methylxanthines at least 7 per week (coffee, tea, chocolate, cola soda, energy drinks). Consumption of energy drinks at least once.
4. Understand and accept the study's procedures and sign an informed consent form.
5. No evidence of somatic or psychiatric disorders as per past medical history and physical examination.
6. The ECG and general blood and urine laboratory tests performed before the study should be within normal ranges. Minor or occasional changes from normal ranges are accepted if, in the investigator's opinion, considering the current state of the art, they are not clinically significant, are not life-threatening for the subjects and do not interfere with the product assessment. These changes and their non-relevance will be justified in writing specifically.

Exclusion Criteria:

1. Not fill the inclusion criteria.
2. Pathological history or evidence of a preexisting condition (including gastrointestinal, liver, or kidney disorders) that may alter the absorption, distribution, metabolism or excretion of drugs or symptoms suggestive of drug-induced gastrointestinal irritation.
3. Present history of a substance use disorder according to Diagnostic and Statistical Manual for Mental Disorders (DSM-V), except for nicotine. Past history of mild substance use disorder (corresponding to substance abuse according to DSM-IV) could be included.
4. Previous or actual psychiatric disorders, alcoholism, abuse of prescription drugs or illegal substances or regular consumption of psychoactive drugs.
5. Having donated blood or having participated in this same study in the preceding 8 weeks, or having participated in any clinical trial with drugs in the preceding 12 weeks
6. Having had any somatic disease or having undergone major surgery in the 3 months prior to inclusion in the trial.

7. Individuals intolerant or having experienced a severe adverse reaction to alcohol or energy drinks. Asian subjects with no intolerance or no serious adverse reactions to alcohol could be included.
8. Having regularly taken medication in the month before the trial, except for vitamins, herb-based remedies, dietary supplements that if, according to the Principal Investigator or his appointed collaborators' opinion, they pose no threat to the subjects and they won't interfere with the study's objectives. Single doses of symptomatic drugs taken during the week before the experimental session will not constitute an exclusion criterion if it can be assumed that it has been completely eliminated on the day of the experimental session.
9. Smokers of >5 cigarettes/day
10. Consumption of >20 g/day of alcohol (females) or of >40 g/day (males)
11. Daily consumption of more than 5 coffees, teas, cola drinks or other stimulant or xanthine-containing beverages in the 3 months prior to inclusion in the study.
12. Subjects unable to understand the nature, consequences of the study and the procedures requested to be followed.
13. Subjects with positive serology to Hepatitis B, C or HIV.
14. Pregnant, breastfeeding women and those using hormonal contraception,. Those not using an effective contraceptive (i.e. abstinence, intrauterine devices, barrier methods or partner vasectomy).
15. Women with amenorrhea or suffering severe premenstrual syndrome.

3 Supplementary Figures and Tables

3.1 Supplementary Figures

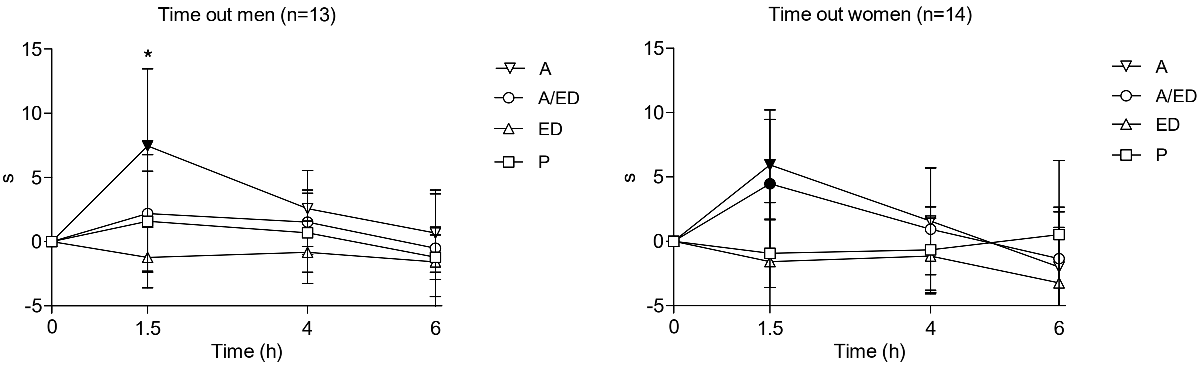

Supplementary Figure 1. Time outside the road in the tracking test.

Data points and error bars represent mean and SD values. A, alcohol+energy drink placebo; A/ED; alcohol+energy drink; ED, energy drink+ placebo alcohol; P, placebo alcohol+placebo energy drink. Differences analyzed with repeated measures ANOVA with treatment and time as factors. *p<0.05; **p<0.01. Filled symbols indicate a significant difference from placebo (p<0.05). The significance is only reported for the comparison of more interest (A *vs*. A/ED) and between all conditions and placebo.

Supplementary Table 2. Time course (T-C) Statistical Analysis

Outcome	Gender	Time-point comparisons and gender differences (ANOVA 3 factors: treatment and time with repeated measures and gender)
**Drunkenness**	Men	A+ED *vs*. P 0.5h* 1-2h** 2.5h*; A+ED *vs*. ED 0.5h* 1-2h** 2.5h*; A *vs*. P 0.5h* 1-2h** 2.5h*; A *vs*. ED 0.5h* 1-2h** 2.5h*
	Women	A+ED *vs*. P 0.5-3h** 4-6h*; A+ED *vs*. ED 0.5-3h** 4-6h*; A *vs*. P 0.5-4h**; A *vs*. ED 0.5-4h**
**Drowsiness**	Men	A+ED *vs*. P 4h*
	Women	A+ED *vs*. ED 2.5h* 4h*; A *vs*. P 4h* 6-8h**; A *vs*. ED 2.5-6h** 8h*; ED *vs*. P 2.5-3h*; A+ED *vs*. A 8h*
**Dizziness**	Men	A *vs*. P 1.5h*; A *vs*. ED 1.5h*
	Women	A+ED *vs*. P 1h* 1.5-2.5h** 4h*; A+ED *vs*. ED 1-2.5h** 4h*; A *vs*. P 1-2h* 3h*; A *vs*. ED 1-2h* 3h*
**Headache**	Men	---
	Women	A *vs*. P 8h**; A *vs*. ED 8h**; A+ED *vs*. A 8h*
**EAVc1**	Men	A+ED *vs*. P 1.5h** 4h*; A+ED *vs*. ED 1.5h** 4h*; A *vs*. P 1.5**; A *vs*. ED 1.5**

	Women	A+ED *vs*. P 1.5-6h**; A+ED *vs*. ED 1.5-6h**; A *vs*. P 1.5-6h**; A *vs*. ED 1.5-6h**
**EAVc2**	Men	A+ED *vs*. P 1.5h**; A+ED *vs*. ED 1.5h**; A *vs*. P 1.5h**; A *vs*. ED 1.5h**
	Women	A+ED *vs*. P 1.5-4h**; A+ED *vs*. ED 1.5-4h**; A *vs*. P 1.5-4h** 6h*; A *vs*. ED 1.5-4h** 6h*
**EAVc3**	Men	A+ED *vs*. P 1.5h**; A+ED *vs*. ED 1.5h**; A *vs*. P 1.5h** 4h*; A *vs*. ED 1.5h** 4h*
	Women	A+ED *vs*. P 1.5-4h**; A+ED *vs*. ED 1.5-4h**; A *vs*. P 1.5-6h**; A *vs*. ED 1.5-6h**
	M *vs*. W	A+ED 4h* M>W; A 6h* M>W
**EAVc4**	Men	A+ED *vs*. P 1.5-4h**; A+ED *vs*. ED 1.5-4h**; A *vs*. P 1.5-4h**; A *vs*. ED 1.5h-4h*
	Women	A+ED *vs*. P 1.5-4h**; A+ED *vs*. ED 1.5-4h**; A *vs*. P 1.5-6h**; A *vs*. ED 1.5-6h**
**BAES-A**	Men	A+ED *vs*. P 1-3h** 4h*; A+ED *vs*. ED 1-1.5h**; A *vs*. P 1-1.5h**; A *vs*. ED 1h*
	Women	A+ED *vs*. P 1-3h**; A+ED *vs*. ED 3h* 4h**; A *vs*. P 1h** 1.5h* 4h*; A *vs*. ED 1h* 4h*
**BAES-S**	Men	A+ED *vs*. P 1-1.5h* 3-4h**; A+ED *vs*. ED 3h* 4h**; A *vs*. P 1h** 1.5h* 4h*; A *vs*. ED 1h* 4h*
	Women	A *vs*. P 2h* 3-6h** 8h*; A *vs*. ED 2h* 3-8h**

**ARCI-PCAG**	Men	A *vs*. P 4h*; A *vs*. ED 1.75h*
	Women	A+ED *vs*. P 4h** 6h*; A+ED *vs*. ED 4h-6h**; A *vs*. P 4h-8h**; A *vs*. ED 1.75h* 4h-8h** ; A+ED *vs*. A 8h*
	M *vs*. W	A 4h* W>M 6h* W>M
**ARCI-MBG**	Men	A+ED *vs*. P 1-1.75h**; A+ED *vs*. ED 1h** 1.75h*; A *vs*. P 1h**; A *vs*. ED 1h**
	Women	A+ED *vs*. P 1-1.75h**; A+ED *vs*. ED 1-1.75h**; A *vs*. P 1-1.75h**; A *vs*. ED 1-1.75h**
**ARCI-BG**	Men	---
	Women	A *vs*. ED 4h*
**ARCI-A**	Men	A+ED *vs*. P 1h*; A+ED *vs*. ED 1h**; A *vs*. P 1h**; A *vs*. ED** 1h
	Women	A+ED *vs*. P 1h** 1.75 h** 4h*; A+ED *vs*. ED 1h**; A *vs*. P 1h** 1.75h-4h* ; A *vs*. ED** 1h
**Number of gyres**	Men	A *vs*. P 1.5h**
	Women	A+ED *vs*. P 1.5h**; A+ED *vs*. ED 1.5h**; A *vs*. P 1.5h**
**Time out (TT)**	Men	A *vs*. P 1.5h*; A *vs*. ED 1.5h**; A+ED *vs*. A 1.5h*

	Women	A+ED *vs*. P 1.5h*; A+ED *vs*. ED 1.5h*; A *vs*. P 1.5h**; A *vs*. ED 1.5h**
**Errors (TT)**	Men	A *vs*. ED 1.5h*; A+ED *vs*. A 1.5h*
	Women	A+ED *vs*. ED 1.5h*; A *vs*. ED 1.5h* 4h**
**Mean latency (PVT)**	Men	A+ED *vs*. P 4h*; A+ED *vs*. ED 1.5h*; A *vs*. P 1.5-6h**; A *vs*. ED 1.5-6h**; A+ED *vs*. A 1.5h* 6h*
	Women	A+ED *vs*. P 1.5h*; A+ED *vs*. ED 1.5-4h**; A *vs*. P 1.5-4h** 6h*; A *vs*. ED 1.5-6h**; A+ED *vs*. A 4h* 6h**
**Median latency (PVT)**	Men	A+ED *vs*. P 1.5h**; A+ED *vs*. ED 1.5h** 4h*; A *vs*. P 1.5-6h**; A *vs*. ED 1.5-4h**
	Women	A+ED *vs*. P 1.5h*; A+ED *vs*. ED 1.5h** 4h; A *vs*. P 1.5-6h**; A *vs*. ED 1.5-6h**; A+ED *vs*. A 1.5-6h**
**Maddox wing**	Men	A+ED *vs*. P 1.5h** 4-6h*; A+ED *vs*. ED 1.5h** 6h*; A *vs*. P 4h**; A *vs*. ED 4h**
	Women	A+ED *vs*. P 1.5h**; A+ED *vs*. ED 1.5h**; A *vs*. P 1.5h**; A *vs*. ED 1.5h** 4h*
**SBP**	Men	A+ED *vs*. P 1h**; A *vs*. P 0.5h* 2h* 2.5h** 3-4h* 6h**; A *vs*. ED 1.5h* 2h** 2.5-6h** 8h*; ED *vs*. P 2h*; A+ED *vs*. A 3h** 4h*
	Women	A+ED *vs*. P 1h*; A+ED *vs*. ED 6h* ; A *vs*. ED 2.5h* 3-6h* 8h*; ED *vs*. P 2.5-3h** 4-6h*

	M *vs*. W	P 2.5h* M>W 8h* M>W
**DBP**	Men	A+ED *vs*. ED 4h*; A *vs*. P 2-6h** 8h*; A *vs*. ED 1.5h* 2-2.5h** 3h* 4-6h**; A+ED *vs*.A 8h*
	Women	A+ED *vs*. ED 4-6h*; A *vs*. ED 2-2.5h** 3h* 4-6h**; 8h*; ED *vs*. P 1h* 2.5h*
**Heart rate**	Men	A+ED *vs*. P 0.5-1h* 2h*; A+ED *vs*. ED 1-2h** 3h** 4h* 6-8h**; A *vs*. P 0.5-4h** 8h**; A *vs*. ED 0.5-8h**; A+ED *vs*. A 1.5h*; ED *vs*. P 3h* 6h*
	Women	A+ED *vs*. P 0.5h* 1.5h* 2h** 2.5h* 8h**; A+ED *vs*. ED 1.5h* 2h** 8h*; A *vs*. P 0.5-3h** 6-8h**; A *vs*. ED 0.5-2.5h** 3h* 6-8h**; A+ED vs. A 1h* 3h*
	M *vs*. W	A+ED 3h* M>W; P 2.5h** 3h** 4h* M>W
**Alcohol concentrations**	Men	---
	Women	---
	M *vs*. W	A+ED 1h* M>W 4h* W>M; A 0.5h* 0.75 h** 1h* M>W 2.25h* 2.5h* W>M
**Caffeine concentrations**	Men	A+ED *vs*. ED 1h* 1.5-8h**
	Women	A+ED *vs*. ED 1h* 1.5h-6h**

**Taurine concentrations**	Men	A+ED *vs*. ED 2.5h*
	Women	A+ED *vs*. ED 0.5h*

Post-hoc comparisons between treatments or genders are shown when they were statistically significant. Taurine: n=12 men, Psychomotor performance (TT and PVT outcomes): n=13 men.

A, alcohol+placebo energy drink; A/ED, alcohol+energy drink; ARCI, Addiction Research Center Inventory; ARCI-PCAG, pentobarbital-chlorpromazine-alcohol group; ARCI-MBG, morphine-benzedrine group; ARCI-LSD, lysergic and diethylamide scale; ARCI-BG, benzedrine group; ARCI-A, amphetamine; BAES-A, Biphasic alcohol effects scale activation; BAES-S, Biphasic alcohol effects scale sedation; DBP, diastolic blood pressure; EAVc1, Driving capability; EAVc2, Willingness to drive an ill child to hospital; EAVc3, Willingness to drive a sick friend home; EAVc4, Willingness to drive a friend to a party; ED, energy drink+placebo alcohol; PVT, psychomotor vigilance task; TT, tracking test; SBP, systolic blood pressure; P, placebo alcohol+ placebo energy drink.*p<0.05; **p<0.01.
